# Modular multimodal platform for classical and high throughput light sheet microscopy

**DOI:** 10.1038/s41598-022-05940-2

**Published:** 2022-02-04

**Authors:** Matteo Bernardello, Emilio J. Gualda, Pablo Loza-Alvarez

**Affiliations:** ICFO-Institut de Ciencies Fotoniques, The Barcelona Institute of Science and Technology, 08860 Castelldefels, Barcelona Spain

**Keywords:** Engineering, Optics and photonics, Biological fluorescence

## Abstract

Light-sheet fluorescence microscopy (LSFM) has become an important tool for biological and biomedical research. Although several illumination and detection strategies have been developed, the sample mounting still represents a cumbersome procedure as this is highly dependent on the type of sample and often this might be time consuming. This prevents the use of LSFM in other promising applications in which a fast and straightforward sample-mounting procedure and imaging are essential. These include the high-throughput research fields, e.g. in drug screenings and toxicology studies. Here we present a new imaging paradigm for LSFM, which exploits modularity to offer multimodal imaging and straightforward sample mounting strategy, enhancing the flexibility and throughput of the system. We describe its implementation in which the sample can be imaged either as in any classical configuration, as it flows through the light-sheet using a fluidic approach, or a combination of both. We also evaluate its ability to image a variety of samples, from zebrafish embryos and larvae to 3D complex cell cultures.

## Introduction

Since its development, light-sheet fluorescence microscopy (LSFM) has gained much interest in the biological and biomedical community. In LSFM, images are generated through a plane-based strategy in which only a single slice of the sample is excited per exposure time, and the emitted photons are collected perpendicularly, forming an image. This procedure is then repeated for each plane of the sample’s volume, leading to optical sectioning and 3D reconstruction capabilities^1^. LSFM therefore provides low induced photodamage, fast acquisition rates, and resolution comparable to traditional confocal microscopy. These advantages made it a necessary tool for the biological and biomedical community, which consequently led researchers to develop several LSFM configurations, each adapted to solve particular experimental needs^2^.

The main efforts for improvements on LSFM technologies were made to achieve higher spatial resolution^3,4^, to increase even more its acquisition speed^5,6^, and to image the *in-toto* volume of the specimen^7–9^. Still, a conventional LSFM platform remains restricted in its imaging throughput capability, mostly because of tedious and time-consuming protocols for optical alignment and sample mounting that are normally embedded in a semi-rigid medium.

This constraint limits the usage of LSFM in highly relevant fields such as high-throughput (HT) studies and drug screenings, in which a high number of samples must be analysed to cover all possible variables and to provide statistical significance. For this reason, in the last years there have been attempts to increase the imaging throughput of LSFM setups for specific applications. While initial solutions aimed at introducing more samples in the embedding medium^10,11^, various subsequent designs pointed to conserve the well-established approach in biology in using multiwell plates. In order to use those plates, the LSFM researchers either adapted the illumination-collection strategy to a single objective, or modified the multiwell plate itself to provide the needed orthogonality between excitation and detection planes^12–15^.

Other approaches have been presented that aim at merging LSFM to flow cytometry-like technologies^16,17^. Here, the main principle is to use microfluidic circuits to flow the sample in a controlled way through a static light sheet. In this case, the different optical sections are captured as the sample goes through the light sheet. This LSFM modality has been used to visualize small samples such as fluorescent particles, phytoplankton and single cells^18–20^. Further evolutions have resulted in compact single microfluidic chips containing also the illumination optics. Such systems have been demonstrated by imaging fixed spheroids and fixed *Drosophila* embryos^21–23^. Additionally, a fluidic-based LSFM system, based on the use of Fluorinated Ethylene Propylene (FEP, n_FEP_ = 1.33) tubes has also been developed. In this case, the FEP tube is used to flow big samples such as 3D cell aggregates, zebrafish embryos, and zebrafish larvae in a HT fashion^24^.

Although effectively increasing the imaging throughput capabilities of LSFM, all these fluidic approaches, however, suffer some drawbacks. Firstly, the acquisition is limited to one or two channels, as the specimen passes only once through the light sheet. Secondly, due to the inner aperture of the fluidic channel, the sample crosses the light sheet with a random angular offset, preventing some features of interest to be efficiently imaged. This is especially important in the case of highly scattering samples such as spheroid, embryos, and larvae, in which it might be interesting to visualize specific structures or organs. In order to image fluorescent neutrophils in live zebrafish larvae, a fluidic circuit was used in which the samples were stopped in the detection field of view (FoV)^25^. Still, no rotation control of the specimen was implemented, and the use of glass capillaries would inevitably generate refractive index mismatches in both the illumination and detection paths, limiting also the resolution power. For the various fluidic approaches listed above, the random positioning of the sample within the fluidic channel makes it difficult to repeat the imaging of the same specimen over time, e.g. in order to visualize in a time-lapse fashion how a particular feature evolves, with an enhanced throughput approach.

Furthermore, while biological and biomedical researchers need to deal in a regular manner with the ability to perform complex experiments, and imaging different type of samples at different spatio-temporal scales, most of the LSFM setups are designed for a specific application. It would be therefore beneficial for the biological community to rely on a single imaging platform that could perform various experiments, ranging from 3D time-lapse movies of a single specimen over several hours, to 3D volume renderings of many samples.

Here we present the optical design of a flexible LSFM imaging system (Flexi-Selective plane illumination microscope or Flexi-SPIM) capable to be adapted to a large variety of experimental needs, allowing full sample control positioning, and fast, multi-channel, *in toto* and in vivo imaging with HT capabilities. We demonstrate its performances for various experimental conditions ranging from developmental biology, to high-speed recording of functional activity, to HT screenings using a variety of biologically relevant specimens, including live and fixed zebrafish and spheroid models.

## Results

### Modularity of the system

The Flexi-SPIM contains illumination, sample mounting and detection modules. This modularity permits to easily modify the overall imaging procedure of the system, enabling a number of radically different experiments on various samples.

#### Illumination module

The illumination module can generate the light sheet through a cylindrical lens (configuration normally referred to as SPIM^1^) or through the combination of two galvo-mirrors and lenses to scan the focused beam along one direction (i.e. Digitally Scanned Laser Light Microscopy or DSLM^26^). With this design, the light-sheet (LS) can be generated either vertically or horizontally, by either rotating the cylindrical lens or by manually removing the cylindrical lens and electronically controlling the two galvo-mirrors, keeping one static. In addition, dual side illumination is implemented by a duplicated illumination arm, producing a second light sheet that enables a more homogeneous sample excitation^27^. The two co-planar light sheets can be generated either sequentially or simultaneously. The first case is ideal for imaging thick and scattering samples in which an important degradation of the LS occurs, preventing the optimal illumination and imaging at the other end of sample. With this scheme each side of the sample can be sequentially illuminated and imaged, and computationally fused together afterwards after removing the respective blurred image. The double simultaneous illumination scheme can be used on transparent specimens in which the LS degradation can be neglected. The switch between simultaneous and sequential double illumination can be electronically controlled through the use of shutters positioned within both illumination arms. In the DSLM mode, the manual modulation of an iris aperture placed close to the back focal aperture (BFP) of each of the illumination objectives enables to reduce the illumination NA, which in turn elongate the available illumination FoV at the expense of an increased LS thickness. This allows adapting the imaging conditions according to the user needs: full open iris will enable better optical sectioning capabilities, while a smaller aperture can provide larger FOV. By using an illumination objective of NA = 0.13 and by varying the iris aperture from 4 mm to fully open, we characterized the variation in the illumination beam’s profile derived (see Supplementary Fig. 2) and measured the LS thickness. These were: 8.95 µm (R1, iris aperture diameter = 4 mm), 6.89 µm (R2, iris aperture diameter = 7 mm), 6.48 µm (R3, iris aperture diameter = 10 mm), 5.33 µm (R4, iris aperture diameter = 13 mm), 5.17 µm (R5, iris aperture diameter = fully open), being R1 to R5 the identification of the selected iris aperture, from the smallest to the largest. Following the Gaussian beam proprieties, the illumination FoV resulted to vary from approximately 258 to 86 µm, in good agreement with the measured values. These values are valid for the single illumination scheme, and therefore by using the double illumination modality they can be increased by a factor of 2, yielding illumination FoVs from 516 to 172 µm. Consequently, the available FoVs are capable to image sample with different sizes. For example, considering the zebrafish model organism, it is possible to visualize its heart (about 100 to 150 µm), its brain (about 400–500 µm), and even the eggs (about 700 µm). For further explication on the illumination module, see “Methods” section and Supplementary Fig. 2.

#### Detection module

**Figure 1:**
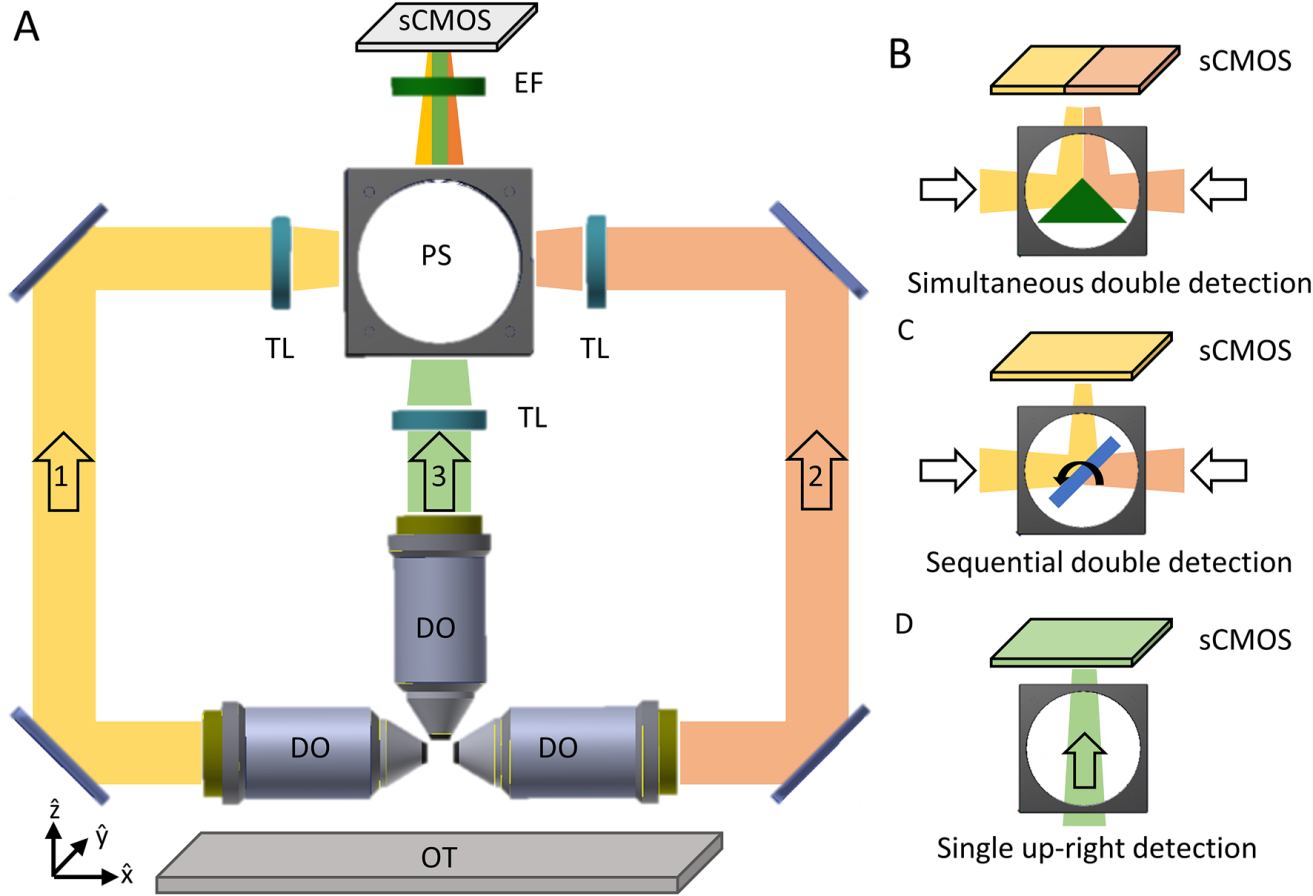
(**A**) Detection scheme enabling the collection of emitted photons through three different paths. *DO* detection objective, *TL* tube lens, *PS* path selector, *EF* emission filter. The coordinate system is indicated respect to the horizontal optical table (OT). (**B**–**D**) The elements included in the PS are switchable and permits obtaining (**B**) simultaneous double, (**C**) sequential double, or (**D**) single detection schemes. All optical paths converge after the path selector to reach the sCMOS camera chip. The FoV determined by the camera chip can be either optically split (B) between the views, or used as a full (**C**, **D**). Figure created with Autodesk Inventor Professional 2015 (www.autodesk.com).

Our design for the detection module makes use of three different objectives. Two of them are located parallel to the optical table and are used when the LS is generated in the vertical direction. The other one, located perpendicularly to the optical table and above the sample holder, is used when the LS is generated in the horizontal direction (Fig. 1A). This allows collecting the fluorescence through multiple optical paths that finally converge at the camera chip to form the image (see below). The three optical paths merge into a Path Selector (PS) module whose elements can be switched as follows: (1) A knife-edge prism to enable simultaneous double side lateral detection (Fig. 1B), using the two horizontally positioned objectives (vertical LS). The prism optically divides the camera chip between the two views, each for each side of the specimen; (2) A motorized mirror to enable sequential double side detection (Fig. 1C) for the same couple of objectives (vertical LS). The rotating mirror allows to use the full camera chip for each of the collecting arms. (3) Finally, no elements are introduced in the PS, resulting in conventional single side detection (Fig. 1D). This using the vertically positioned objective and setting the LS to the horizontal position. The multiple detection scheme allows to fully exploiting the flexibility offered by the illumination module and the different sample mounting systems here presented, while maintaining a single final optical path. Notice that such scheme is compatible with any eventual supplementary detection add-ons (e.g. filters, remote focussing elements, adaptive optics, phase masks, etc.). The final resolution obtained from the optical system depends on the actual objectives and tube lenses used. The design enables using tube lenses having the same focal length (200 mm) for the three paths, obtaining the same magnification/resolution in all the views. Otherwise the lateral paths enable also the insertion of different tube lenses (180 mm). In our experiments we tested detection objectives ranging from 0.3 to 0.5 NA which, in combination with the tube lens and camera specifics, achieve theoretical lateral resolutions from 1.07 µm to about 0.64 µm, i.e. adaptable to various experimental needs. Practically, we measured the lateral resolutions obtained to be of about 1.2 µm and 0.88 µm respectively, which permit the imaging at cellular level of entire organisms (see also “Methods” section and Supplementary Material for the complete characterization).

#### Sample scanning and mounting modules

**Figure 2:**
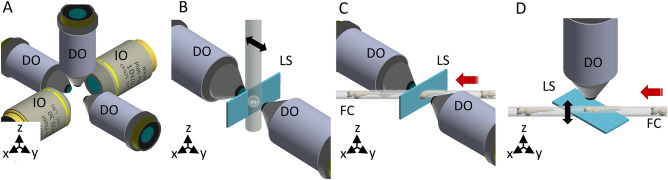
(**A**) The objectives’ configuration enabling multiple illumination/detection schemes. (**B**) Through the first mounting option the sample is embedded within a cylinder perpendicular to the optical table. The black arrow indicates the scanning direction. (**C**) The second approach uses the flow of the samples to scan them through the light sheet. The red arrow indicates the flow direction. (**D**) Through the third approach, the sample flow (red arrow) is used to position the sample in the objective’s FoV of the DO. The scanning of the specimen is obtained instead in the vertical direction (black arrow), either mechanically or optically. *IO* illumination objective, *DO* detection objective, *LS* light sheet, *FC* fluidic circuit. Figure created with Autodesk Inventor Professional 2015 (www.autodesk.com).

One of the key features of our platform is the different possibilities for the sample mounting, which is a critical factor in LSFM as it can limit or boost the performance of the entire system. In order to provide flexible, fast and easy-to-use sample’s loading capabilities, we designed three different interchangeable sample mounting approaches (Fig. 2).

The first scanning option reflects what is normally considered a classical approach, in which the sample (usually embedded in agarose forming an optically clear cylinder) is placed to cross the LS, enabling a double side illumination/detection^9^ scheme (Fig. 2B). Volume scanning is achieved through the sample’s displacement and/or rotation.

The second scanning approach makes use of a fluidic circuit to mount the specimen. The circuit is based on a Fluorinated Ethylene Propylene (FEP) tube. FEP is a material that presents a refractive index of about 1.33, hence an excellent match for water-based imaging medium, allowing the use of water-dipping objectives and minimise optical aberrations^28^. The fluidic circuit crosses the water-filled imaging chamber at 45° (Fig. 2C) and it is connected to a flow controller^24^. Once the sample is loaded in the circuit, it can be displaced at a constant flow rate. This approach permits to use the continuous sample flow as the mean for volume scanning across a static vertical light-sheet.

The third mounting approach uses the fluidic circuit only to position the sample in the light sheet region. Once it is there, the sample’s flow is stopped and the specimen can then be scanned across the LS (in this case set to the horizontal position). This is done either mechanically, translating the full chamber with the FEP tube containing the specimen, or optically, through the LS displacement mediated by one galvanometric mirror in synchrony with the movement of the collection objective using a piezo scanner^29^ (Fig. 2D). In addition, as explained in the following section, the FEP tube can be rotated around its longitudinal axis, enabling to better expose a particular feature of the sample, that otherwise would be hidden by the specimen itself, toward the detection objective (e.g. to image the heart or the brain of the zebrafish). Notice that this mounting scheme would also enable remote focusing using an electrically tunable lens or other wavefront coding strategies^5,6^.

Importantly, and opposite to traditional mounting schemes, the use of fluidic methods allows to consecutively load different samples. Therefore, specimens can be mounted and imaged in their own culture media, helping in maintaining adequate physiological conditions. After imaging, these samples can be placed back in the culture medium or incubator for future inspections without any further protocols (such as the removal of solid agarose). This approach therefore increases both the sample-throughput and the subsequent specimen viability. In addition, by stopping the sample under the objectives’ FoV, our third method allows easy sample loading and imaging of the developing specimen and their selected features for long periods of time. It therefore combines the advantages of the two previously described approaches.

### Multi-modality of the system

Through the combination of the modules illustrated above, three different imaging modalities are resulting, each of them with particular advantages. The imaging modalities are here called *Classic* LSFM, *Flow* LSFM, and *Hybrid* LSFM. While the first configuration is ideal for long-term imaging, the second allows increasing the throughput of the system. Finally, the third configuration can be used for both long-term imaging and for enhanced throughput, also assuring the repeatability of the imaging session. Therefore, through the implementation of a single optical setup, called Flexi-SPIM (see also Supplementary Fig. 1–3 and Supplementary Tables 1 and 2 for a complete description), a variety of experiments can be performed, as explained below.

**Figure 3:**
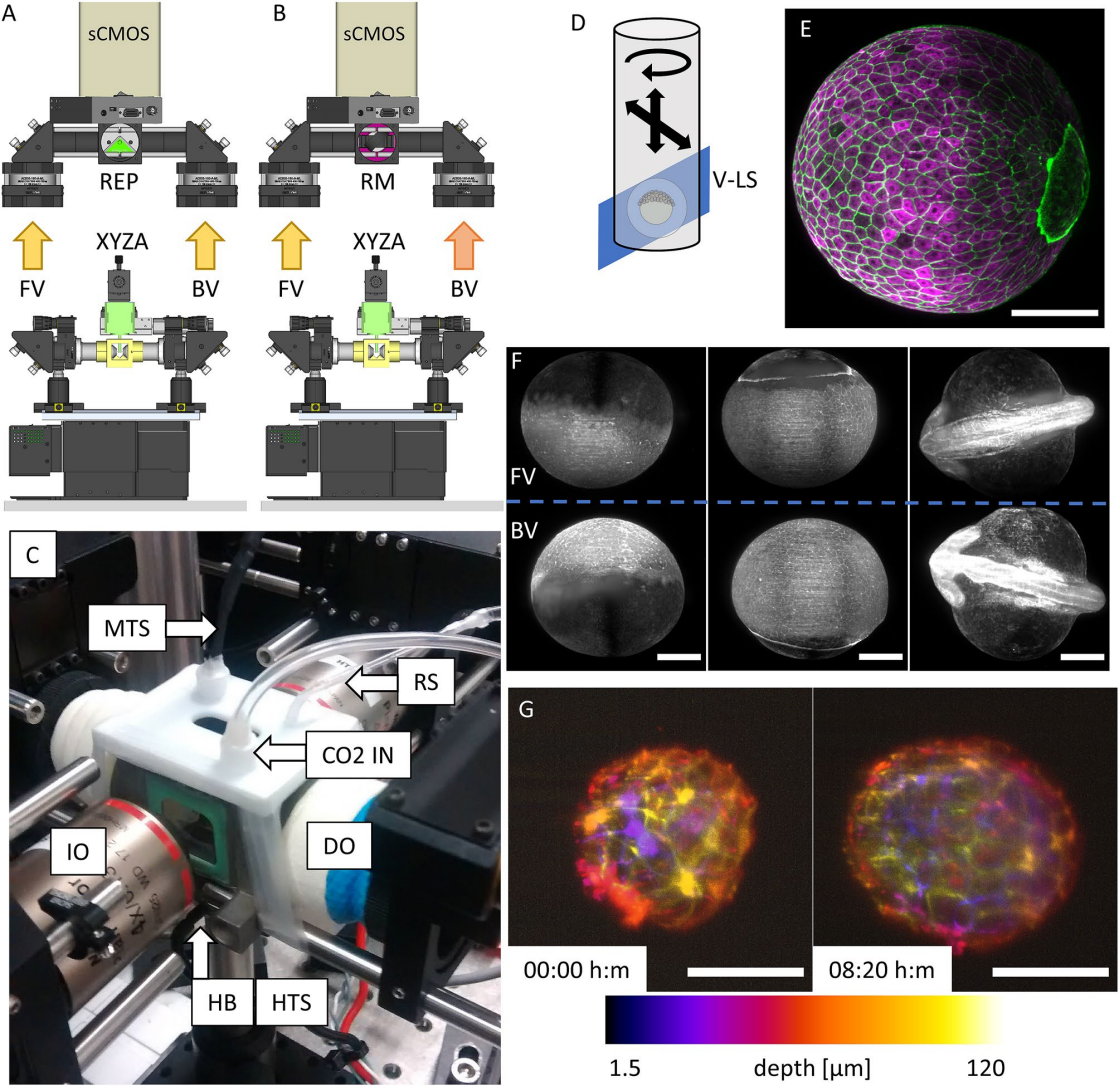
(**A**, **B**) The setup implementation in the Classic LSFM modality, for (**A**) simultaneous and (**B**) alternate double side detection (FV: front view, BV: back view), using a sCMOS scientific camera (sCMOS). The path selector is a right-edge prism (REP) in (A) and a rotating mirror (RM) in (**B**). (**C**) Photo of the 316L steel imaging chamber, with the two air illumination objectives (IO) and the two sealed water detection objective (DO). *MTS* medium temperature sensor, *RS* refilling system, *CO2 IN* CO_2_ inlet, *HB* heater block, *HTS* heater temperature sensor. (**D**) Exemplification of the sample mounting and scanning system. The sample position can be controlled in 3D and rotated (XYZA) around its vertical axis. *V-LS* vertical light-sheet. (**E**) Maximum intensity projection of a fixed zebrafish embryo at 90% epiboly, labeling actin (green) and tubulin (magenta), showing multicolor capability and achieving homogenous excitation thanks to the double illumination scheme. Scale bar 200 µm. (**F**) Maximum intensity projections from 3 time points of a time lapse movie recording an unconstrained developing zebrafish embryo (actin-GFP) from blastula to segmentation stages. The REP was used to register the front (top) and back (bottom) views onto the same camera chip, at the same time. A double illumination scheme was used for excitation. Scale bars 200 µm. (**G**) Depth colored intensity projection of an E-Cadherin-GFP mouse embryo at E4.5 stage (left) and after about 8 h (right) of imaging. Temperature and pH were conditioned within the imaging chamber, enabling cells proliferation and embryo’s development. The entire volume of the mouse embryo has been imaged every 10 min, through the RM, i.e. alternating the detection of front and back views. The showed images are obtained from the fusion of the two views. Scale bars 100 µm. (**A**, **B**) created with FreeCAD 0.16 (www.freecadweb.org) and (**D**) created with Autodesk Inventor Professional 2015 (www.autodesk.com).

The *Classic LSFM* follows the traditional design of SPIM or DLSM setups, and makes use of two illumination objectives and two detection objectives, allowing a dual side excitation/collection scheme. The four objectives lay on the horizontal plane, around a custom-designed imaging chamber. While the detection is implemented through water-dipping objectives, sealed within the chamber, the excitation vertical LS is generated by the two air-based objectives, passing through two coverslips glued to the chamber (Fig. 3). The imaging chamber is 3D printed in medical grade stainless steel (316L, from Materialise Manufacturing Services) and it also integrates a heating resistor, managed by a proportional–integral–derivative (PID) controller, and a continuous flow of CO_2_. This combination enables the autoclavability of the chamber, besides the regulation of temperature and pH of the imaging medium. Thanks to this environmental control, we were able to maintain the necessary conditions for imaging delicate live specimens during prolonged time periods: up to 8 h for E4.5 mouse embryos, and up to 24 h for zebrafish embryos at different developmental stages. The 3D scanning is performed through mechanical translation of the sample through the static vertical LS, by means of a linear stepper motor stage. An additional stepper motor rotational stage is also included, to enable the rotation of the sample respect to its vertical axis (Fig. 3D).

As the system provides a double detection scheme, by inserting in the path selector of the collection module the knife-edge prism mirror (Fig. 3A) it is possible to combine the frontal and back views (Fig. 3F) of the sample during the same exposure time. Alternatively, when the knife-edge prism is substituted by the mirror mounted on a stepper motor (Fig. 3B), it is possible to use the full FoV of the camera for each of the detection objectives, and thus use objectives with higher magnification or numerical apertures. In such case, the sample would be displaced through the LS and the alternate selection of the front and back views can be used for visualising the front/back of the sample (Fig. 3G).

One of the main applications of this configuration involves developmental biology, in which particular structures of interest are recorded for a long period of time. Particularly, we have recorded the development of zebrafish embryos trough nuclei, actin, or microtubule staining, from cleavage stages to gastrulation and beyond, obtaining the 3D structure of the entire sample volume. Using a sequential double side illumination and a double side lateral detection scheme (with Nikon 10×, 0.3NA objectives) we can record both half-volumes of the embryo simultaneously on the same camera chip (2048 × 2048 pixels) (Fig. 3F and Supplementary Video 1). The images obtained from both sides are then fused on a single dataset using fusing algorithms^30^ (Supplementary Video 2). Multicolour and time lapse images of fixed (Fig. 3E) and living samples are also possible (Supplementary Video 3).

**Figure 4:**
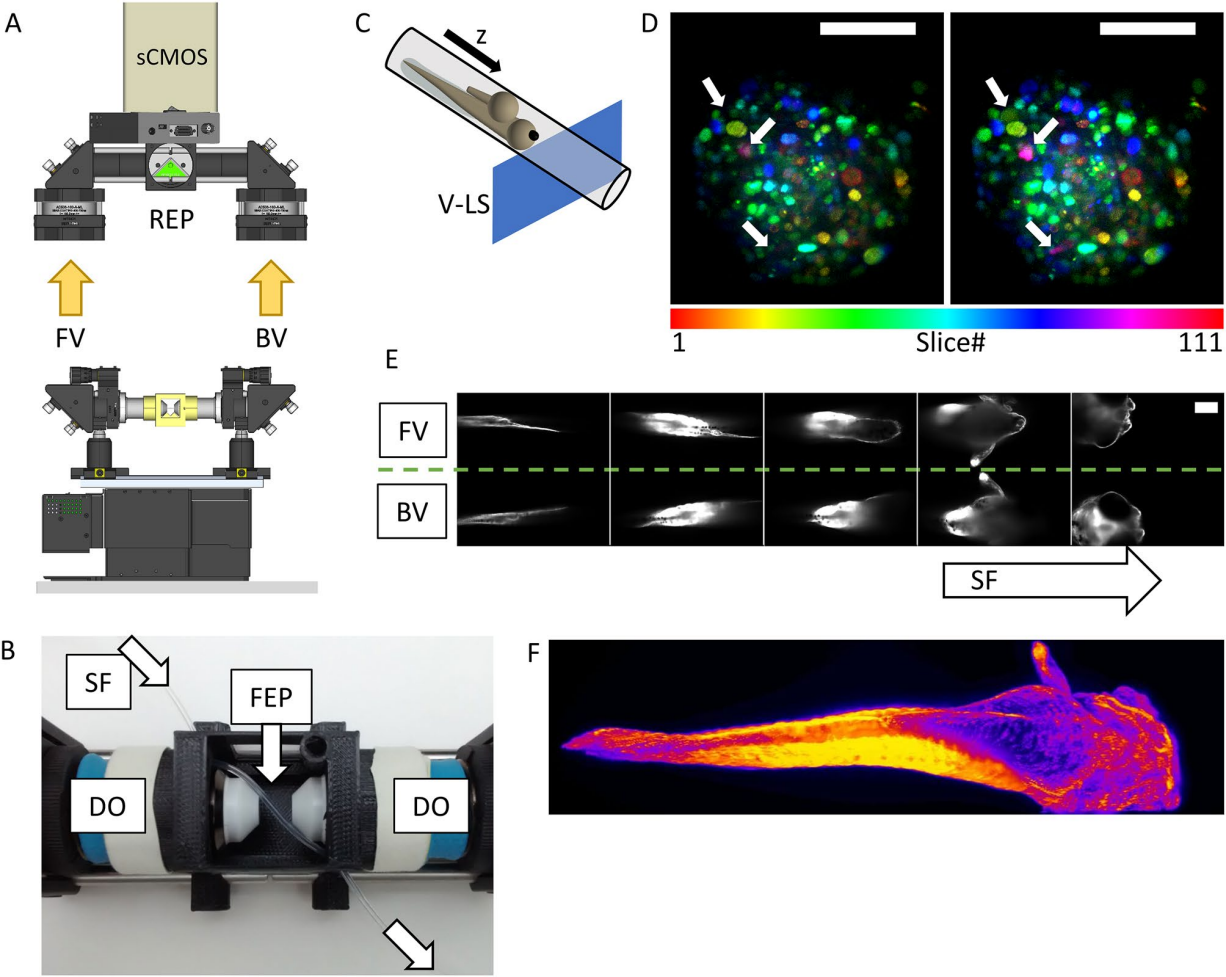
(**A**) The setup implementation in the Flow LSFM modality with double simultaneous detection scheme. (**B**) The imaging chamber embedding two sealed water detection objective (DO) and a FEP tube crossing it at 45°. *SF* sample flow. (**C**) Exemplification of the sample mounting and scanning system. The sample flows within the FEP tube through a vertical light-sheet. (**D**) Depth-colored maximum intensity projection of a fixed tissue spheroid expressing a histone H2B fluorescent nuclear reporter protein (mCherry), obtained through single (left) and double (right) side detection scheme. The fusion of two views (on the right) reveals nuclei that would be otherwise missed through single side detection (see white arrows). Scale bars 100 µm. (**E**) Series of images during the flow of a Fli-GFP zebrafish embryo through the system. The front view (FV) and the back view (BV) are simultaneously registered on the same camera chip. Scale bar 100 µm. (**F**) 3D reconstruction view of the same zebrafish showed in (**E**). Colors represent signal intensity (Fire LUT in Fiji). (**A**) created with FreeCAD 0.16 (www.freecadweb.org) and (**C**) created with Autodesk Inventor Professional 2015 (www.autodesk.com).

Substituting the previous imaging chamber with a custom-designed 3D printed cuvette crossed by a FEP tube, we can switch from the classical modality to what we call the *Flow LSFM* mode (Fig. 4A, B). As previously, four objectives laying on the horizontal plane are employed: two air-objectives for the illumination through a vertical LS, and two water-objectives sealed within the chamber, for the emission collection. In order to assure the optical access and at the same time intersect all objective’s foci, the FEP tube crosses the chamber on the horizontal plane at 45° with respect to the light sheet. As explained above, the sample is loaded into the FEP tube using a syringe pump which allows the control of the position and flow speed of the specimen. This also allows programming capabilities of protocols for sample unloading and retrieving it again if necessary. While the sample is continuously flowing through the vertical LS (Fig. 4C), the two detection objectives retrieve the views of its 3D structure. Both views are then projected onto the camera chip at the same time through the knife-edge prism mirror. This modality enables whole-sample high-throughput screens possibilities, thanks to the easiness and fast sample loading, resulting in an effective high-resolution 3D imaging flow-cytometer system. For thick and scattering samples, the double side illumination enables a homogeneous excitation of the fluorophores while the double simultaneous detection scheme permits to retrieve features that would be otherwise hidden by the sample volume itself, as shown in Fig. 4D. The same principles can be used to reconstruct the entire volume in 3D of much bigger samples such as entire and elongated zebrafish embryos (Fig. 4E, F) that would not fit in the FoV of conventional LSFM schemes. Moreover, for thin and transparent samples the record of multichannel images can be done by inserting two different emission filters in the two detection paths. In this case, the knife-edge prism permits the recording of the two channels on the same camera chip (not shown).

**Figure 5:**
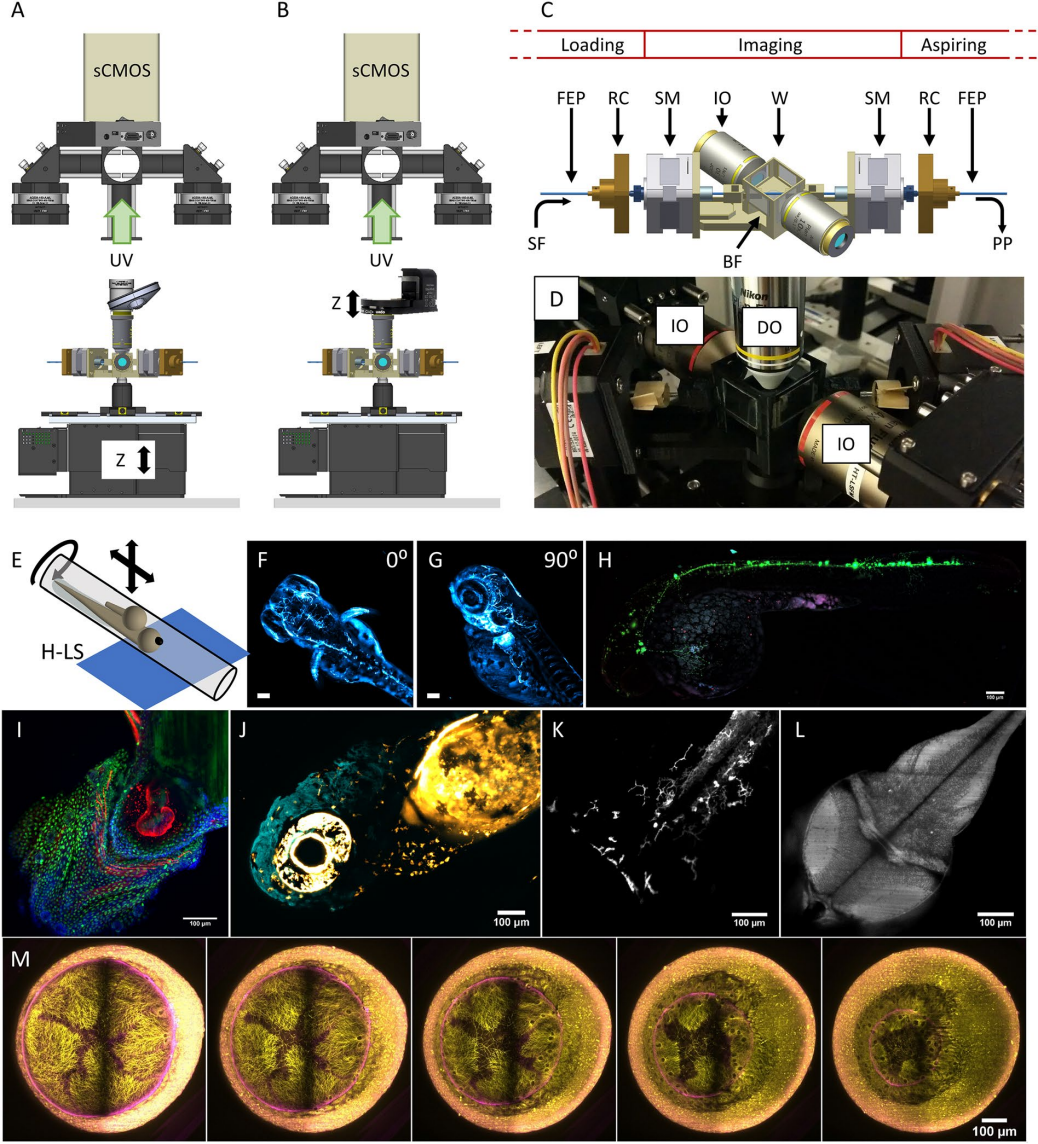
Setup implementation in the Hybrid LSFM modality with upright detection scheme and examples. The scanning in the depth (z) direction can be performed either (**A**) through a vertical stage moving the entire chamber or (**B**) through the displacement of the detection objective with a piezo-element synchronized with galvanometric scanning of the light sheet. Schematic (**C**) and actual implementation (**D**) of the imaging chamber for the Hybrid LSFM modality. (**E**) Exemplification of the sample mounting and scanning system. The sample flows within the FEP tube until the objectives’ FoV. There, it can be rotated around the FEP tube’s axis to orient the sample correctly (as shown in **F**, **G**) and/or it can be moved in a controlled and stepwise manner along the tube, providing multichannel high-resolution whole larvae imaging by stitching (**H**). (**I**) The system allowed high-throughput imaging of zebrafish heart in a single imaging session (18 samples. The showed image was processed through a background correction method to decrease yolk’s auto-fluorescence). Full set of images in Supplementary Fig. 7. The use of a piezo focus control (**B**) allows fast imaging of macrophage migration in the head (**J**) and the caudal fin (**K**) as well as zebrafish brain’s neural activity using calcium indicators (**L**). More information in Supplementary Videos 5, 6 and 7. (M) Long-term multicolor time-lapse movies of zebrafish embryo development are also achieved. Scale bars: 100 µm. *UV* upper view, *SF* sample flow, *RC* rotational connector, *SM* stepper motor, *W* water compartment, *PP* programmable pump (not shown), *BF* bright field compartment, *IO* illumination objective, *DO* detection objective. (**A**, **B**) created with FreeCAD 0.16 (www.freecadweb.org) and (C, E) created with Autodesk Inventor Professional 2015 (www.autodesk.com).

Using the same sample loading approach through FEP tube, removing the knife-edge prism mirror and setting the light sheet to horizontal, we can convert the detection path to a single upright detection scheme (Fig. 5A, B). This is part of the so-called *Hybrid LSFM* mode, in which samples are loaded and brought within the field of view of the camera using the syringe pump (fluidic loading) but imaged in a classical way.

The fluidic circuit is composed by three consecutively connected FEP tubes within a third 3D printed imaging chamber: the “loading-tube” in which the sample is loaded, the “imaging-tube” that crosses the chamber, and the “aspiring-tube” that is connected to the pump (Fig. 5C–E, and Supplementary Fig. 4). The two air-objectives generate the horizontal LS, while a single vertical water-objective is dipping within the medium compartment of the chamber, for the collection of the emitted photons (Fig. 5D). The imaging proceeds as follow: the sample and medium are inserted into the “loading-tube”, and aspired by the syringe pump towards the “imaging-tube” until reaching the field of view of the detection objective. Once here, the specimen is automatically detected, and its flow is stopped. At this point, two stepper motors integrated in the imaging chamber can rotate the “imaging-tube”, rotating also the sample to align it in a desired direction and to expose a particular feature of interest toward the detection objective (Fig. 5F, G, and Supplementary Fig. 4). The sample’s volume can now be scanned in two ways. The first possibility consists on translating the entire imaging chamber in the vertical direction through a vertical stage across the static horizontal light sheet(s) illuminating the focal plane of the detection objective. The second option consists on the refocusing of the detection objective through a piezo element, which is in turn synchronized with the galvo scanner in the illumination path^29^. In that case the sample remains static while the light-sheet(s) moves synchronously with the detection objective’s focal plane. In our implementation, this approach allowed to reach acquisition rates of 0.5 and 1 volumes/second (typically 1 vol is made of 100 optical sections, varying the z-step size depending on the sample thickness).

Moreover, we have integrated a LED in the chamber that allows obtaining bright-field images. These are critical for the detection of the passage of the specimen, stop it under the objective’s FoV and to adjust the specimen orientation. Additionally, we can acquire a stack of bright-field images and use stack focusing algorithms to keep only the in-focus information of the different planes (Supplementary Fig. 5), obtaining a high-quality bright-field imaging.

The *Hybrid LSFM* approach presents several advantages. Compared with agarose-based C*lassic LSFM*, the loading of samples is extremely facilitated, being based on the use of the syringe pump and the FEP tubes, increasing at the same time the throughput capabilities of the system in a natural way. Secondly, compared to the *Flow LSFM* mode, it easily allows multicolour and repeatable imaging of a large population of samples. It also enables to image long samples, such as entire zebrafish larvae that exceed the available field of view, by stitching (Fig. 5H). In that case, the syringe pump performs the translation to the next sample’s section to be imaged. Although the detection makes use of a single objective, the designed imaging chamber allows to rotate the sample in order to obtain multi-view recording, or to expose the part of interest of the sample toward the objective. This is particularly useful for heart, head, caudal fin, or brain imaging of zebrafish larvae (Fig. 5I–L), which views might be hindered from a non-controlled orientation of the specimen. Finally, the *Hybrid LSFM* mode allows performing 3D time-lapse movies in different conditions, such as 3D multicolour dual side illuminated movies of the macrophage movement in zebrafish head and fin (Fig. 5J, K, and Supplementary Videos 5 and 6); neuron activity in the full zebrafish brain at 0.5 volumes/second (50 planes) (Fig. 5L and Supplementary Video 7), or alternate dual side illumination multicolour imaging of zebrafish embryo development (Fig. 5M). Thanks to all the new capabilities offered by the *Hybrid LFSM* mode (facilitated sample loading, 3D multicolour stack recording, and automated sample positioning) we have been able to perform volumetric HT imaging in a single day of ~ 100 complex 3D-3 culture models^31^ including a co-culture of tumour cell spheroids of non-small cell lung carcinoma, cancer associated fibroblasts and a monocytic cell line (THP-1) on alginate capsules (Supplementary Fig. 6), or tens of three-colour fixed and immunostained zebrafish embryos (Supplementary Fig. 7). Additionally, more complex experiments in which the imaging of various samples can be repeated before and after a defined interaction (e.g. to obtain a control image^32^, or the addition of a drug) are now possible, thanks to the easiness of sample’s loading and unloading procedures that maintain sample’s viability. Note that in the *Hybrid LSFM*, as in the *Flow LSFM*, the medium that fills the FEP tube can be the medium that best fit the experimental need (e.g. culturing media for organoids, or E3 medium for zebrafish, or medium with the addition of drugs for chemical interaction experiments). A summary of the main features of the different acquisiton modes available within the Flexi-SPIM setup is shown in Table 1.

**Table 1:** Summary of the different acquisiton modes available within the Flexi-SPIM setup.

Acquisition mode	Dual side illumination	Dual side detection	Light sheet orientation	Multi-colour	Sample rotation	Mounting	Optimized for	Drawbacks
Classic LSFM	Yes	Yes	Vertical	Yes	Yes	One by one (agarose)	3D Time-lapse, multicolour imaging	Low throughtput
Flow LSFM	Yes	Yes	Vertical	Possible	No	Multiple samples within FEP tube	Low resolution, increased throughput	Single colour on thick samples
Hybrid LSFM	Yes	No	Horizontal	Yes	Yes	Multiple samples within FEP tube	High resolution, increased throughput, multicolour imaging, stitching	Single side detection

### Additional capabilities

An additional add-on for the Flexi-SPIM platform is a 3D printed multiwell plate reader (MPR) system, which accepts common 96-multiwell plates and is connected to the distal tip of the FEP “loading-tube” (fully explained in Supplementary Fig. 9). The MPR aims to move the tip of the loading-tube over the plate containing multiple samples and insert it into the desired well. Once the tip is within the well, the syringe pump will aspire the specimen transporting it toward the FoV. The combination of the MPR, the motorized imaging chamber, and the *Hybrid LSFM* approach, permits not only the full automation of the sample loading, imaging, and sorting procedures of multiple samples but also the definition of automated drug delivery to the specimen directly in the multiwell plate. This also thanks to the automation of communications between of all the devices through an in-house implemented LabVIEW-based program.

Finally, we also tested the possibility of adding new contrast mechanisms to our system, such as Raman LSFM^33^. In here, light sheet illumination is performed with a laser operating at 637 nm. On the detection path an interferometric tunable filter (TF) mounted on an Arduino-controlled stepper motor is inserted before the tube lens in the *Hybrid LSFM* mode arm (Supplementary Fig. 10). By changing its orientation angle the central wavelength can be continuously tuned, maintaining its bandwidth. This allows recording on every pixel a cumulative intensity distribution of the vibrational Raman bands as the TF angular position is changed. Subsequently, a simple differentiation of the images is used to recover the Raman spectrum. Since this procedure is performed pixel-by-pixel on the entire image, a 2D spectrally resolved Raman map is obtained. We tested our LSFM Raman module measuring the characteristic Raman spectra of ethanol, methanol and water. The obtained Raman spectra are in good agreement with the spectra taken under a confocal micro Raman system. Although the spectral resolution of our approach might be improved through narrower TF, the image acquisition speeds up the Raman mapping making it suitable for quick inspections of 3D samples^33^.

## Discussion

Biological and biomedical research often need to deal with complex specimens whose development, functions, or drug-induced effects are not trivial to understand. LSFM offers unprecedented high x, y, and z resolution and optical sectioning capabilities along with low photobleaching rates and high-speed imaging. However, the big variety of experiments needed to fully understand the biological complexity spans over different spatial and temporal scales and it often involves the statistical evaluation of many specimens. This diversity cannot until now be practically addressed by using the current imaging approaches. Our LSFM here demonstrated has a straightforward and automated sample mounting scheme that is capable to perform long-term imaging as well as high-throughput screens. As it is based on the LSFM concept, it contains all its inherent advantages. Furthermore, this can be used in a wide range of applications, going from developmental to functional imaging, to high-throughput screenings.

Here, we have described three different highly automatized acquisition modes, which are combined on a single machine: (*i*) *Classic LSFM*, with mechanical sample scanning and sample rotation, (*ii*) *Flow LSFM* with fluidic scanning of the sample using specialized syringe pumps, and (*iii*) *Hybrid LSFM*, which combines fluidic loading of samples and high-speed volume’s scanning. We have also evaluated the performance of the Flexi-SPIM platform and its ability to carry out imaging of large populations in a variety of samples (Supplementary Fig. 6 and Supplementary Fig. 7), from zebrafish embryos and larvae to 3D complex cell cultures, with high resolution, and minimal photo-bleaching. The achieved throughput will depend very much on the specific application and samples, since the main limitation comes from the loading process. The highest throughput is achieved on the complex 3D culture imaging experiment, where several (around 10–20) samples are loaded at once and imaged sequentially in the *Hybrid LSFM*. For this experiment we considered 12 distinct types of 3D cell cultures with a total of 110 specimens imaged in 5 h, which leads to a throughput of about 20 samples per hour. When imaging zebrafish, each specimen needs to be loaded individually. The entire imaging process (loading the sample, positioning it within the FoV, rotating it to face the feature of interest, acquiring a 3D-3colour stack, and discarding a fish) took around 10–12 min, so the throughput was limited to 5 to 6 fish per hour.

The demonstrated system results in a robust and flexible multimodal light-sheet microscopy-based compact platform. The three different modalities could be interchanged in few minutes and the re-alignment is extremely straightforward as changing from vertical to horizontal light sheets can be easily done thanks to the electronically controllable galvo-mirrors. An open source approach using custom-made Arduino-based electronics are used to control the shutters, LEDs, and rotational motors (Supplementary Fig. 8). Importantly, we have also demonstrated an advanced sample loading system that enables extending the throughput capabilities of the previous configuration of LSFM. In addition to that, our modular detection scheme, which uses a single camera, is key for allowing multiple imaging modalities. Finally, in addition to double-sided illumination/detection scheme, our system possesses an additional detection channel (three in total) that is used in the *Hybrid LSFM* configuration.

Samples mounted through the fluidic-based loading system can be easily imaged using water dipping objectives, as in an upright microscope. This approach allows the straightforward interchange of objectives (from 10 × 0.3NA to 40 × 0.8NA), the possibility to combine light sheet imaging with bright-field microscopy (and its variant of “stack focussing”), and the addition of Raman light-sheet functionalities. From the practical point of view, we have also shown examples of a rich variety of experiments and applications (from time-lapse movies to high-throughput screens and 3D volume renders), all of them recorded with the same microscope.

Although the use of syringe-based loading for *Flow LSFM* and *Hybrid LSFM* modes is useful, some precautions need to be taken. Due to its fragility, the loading of zebrafish embryos is advisable to use a manual protocol, i.e. pipetting them into a loading pool and pulling them at low suction speeds. For harder samples, such as adult larvae, complex 3D-cultures or organoids, the multiwell plate loading system could be alternatively used. Moreover, although the rotation of the sample is possible, the sample may slip and not perfectly follow the FEP tube rotation. For that reason, for the *Classic LSFM,* multi-view fusion algorithms can be easily implemented. The idea behind sample rotation in *Hybrid LSFM* mode is not to fulfil this requirement, but to rotate the area of interest of the embryo (heart, brain, eye, etc.) towards the detection objective.

The modularity of our system also permits other functionalities to be easily implemented. On the illumination side Bessel, Airy, or 2-photon excitation can be easily added through the DLSM version^34,35^, to achieve larger FOV and help in the imaging of thicker and highly scattering samples. On the detection module, the port that has been here used for light-sheet Raman microscopy, could also be used to introduce optical manipulation capabilities such as photoactivation or laser ablation. In this way, repeatable LSFM imaging and sample manipulation of a large population would be possible, in a high throughput and fully-automatic approach. This would potentially boost research e.g. in the field of regeneration studies. Finally, the combination of the high-throughput capability with remote focusing or wavefront coding techniques for fast volumetric imaging could also be implemented^5,6^. The openness of the system will allow performing these improvements and the conjugation to new modules that further extend the platform’s capabilities.

## Methods

### Illumination path

Four CW lasers with excitation wavelengths of 405, 488, 561 and 637 nm (Cobolt, MLD 50 mW, MLD 50 mW, DPL 100 mW and MLD 150 mW, respectively) are used for excitation.

On its basic implementation (single side excitation), the illumination arm uses a telescope system composed of two achromatic doublets (Thorlabs, AC254-050-A-ML, f = 50 mm, and AC254-200-A-ML, f = 200 mm) to expand the beams. Subsequently, the air-objective (either Nikon 4 × PlanFluor NA 0.13, or Nikon 10 × PlanFluor NA 0.3) is mounted on a manual translation mount (Thorlabs, SM1Z), allowing fine positioning of the beam waist. At the back focal plane of the objective, a circular slit is added in order to adjust the illuminating numerical aperture and thus, the light-sheet thickness along the camera FoV. Double side illumination is achieved by duplicating the above-described elements and adding a 50/50 beam-splitter cube (Thorlabs, CCM1-BS013) and a relay lens set with two achromatic lenses (Thorlabs, AC254-075-A-ML, f = 75 mm) at the entering of the illumination arms. An Arduino controlled servo motor inserted in each illumination arm works as shutter, allowing the selection of the illumination side. As described before the system can work in SPIM or DSLM configurations. In SPIM configuration, a 150 mm round achromatic cylindrical lens (ACY254-150-A) is mounted on each illumination arm in a cage rotational mount (CRM1). Additionally, an adjustable slit (Thorlabs, VA100C) will allow adjusting the light sheet height at the illuminated plane. In DSLM configuration the light sheet is created by a pair of galvanometric mirrors (Thorlabs, GVSM002). The two galvo mirrors are mounted independently, so perfect conjugation of optical planes will be achieved. Two achromatic doublets (Thorlabs, AC254-030-A-ML, f = 30 mm) projects the pivoting beam from the first onto the second galvo mirror. Two identical achromatic doublets perform the same operation between the second galvo mirror and the centre of the 50:50 beam splitter cube, which in turn results conjugated with the back focal aperture of the illumination objectives. The DSLM module could be easily attached to the SPIM illumination block using four rod adapters (Thorlabs, ERSCA). The whole block is supported with a mounting post (Thorlabs, P200) and a clamp system (CH1530/M), ensuring stability and modularity. All deflections of the illumination beams are performed through elliptical mirrors (Thorlabs PFE10-G01) mounted in right-angle cage mounts (Thorlabs, KCB1E). For Raman excitation, we use two filters (Semrock, FF01-731/137–25) in order to filter the illumination wavelength of the 637 nm laser. More information on the implementation of the illumination module can be found in Supplementary Fig. 2.

### Detection path

Different detection objectives are compatible with our design, both water dipping (Olympus UMPLFLN10XW NA 0.3, UMPLFLN20XW NA 0.5, or Nikon 10 × 0.3) or air objective (Nikon 10 × 0.3).

On each detection arm an achromatic doublet (AC508-200-A-ML, f = 200 mm, or AC508-180-A-ML, f = 180 mm depending on the objective brand, and AC254-200-A-ML, f = 200 mm for Hybrid Mode arm, from Thorlabs) is used as tube lens. In the path selector component, implemented through a cage cube (C6W, Thorlabs), the element inserted is either a knife-edge prism (MRAK25-P01, Thorlabs), a rotating mirror (PFR10-P01 Thorlabs on a Nanotec, L4018S1204-M6 stepper motor), or no elements. After the path selection, the collected radiation is passing through the emission filter (either 520/15, 590/50, 638LP, from Chroma and Semrock) inserted into a motorized filter wheel (FW102C, Thorlabs). Subsequently, the images form onto the chip of a Hamamatsu Orca Flash4.0 sCMOS camera. For the Raman module a tunable filter was used (Semrock, TBP01-790/12) mounted on an Arduino controlled stepper motor (Nanotec, L4018S1204-M6). All deflections of the collected beams are performed through elliptical mirrors (Thorlabs PFE10-G01) mounted in right-angle cage mounts (Thorlabs, KCB1E). More information on the implementation of the detection module can be found in Supplementary Fig. 3.

### Sample scanning

Depending on the chosen modality, sample scanning is performed differently. When samples are mounted in the *Classic LSFM* configuration, the embedding cylinder is attached to a linear stepper motor stage (Thorlabs, LNR25ZFS) for sample translation across the light-sheet plane, and an Arduino controlled stepper motor (Nanotec, L4018S1204-M6) for sample rotation. When the *Flow LSFM* mode is used, samples are loaded into the FEP tube (Adtech Polymer Engineering Ltd.) circuit and flowed through the light sheet using a syringe pump (Tecan, Cavro Centris). The used specialized and programmable syringe pump permits to set the needed flow rate, from 5 ml/s until 5 nl/s, and the quantity of liquid displaced with a resolution of 1.4 nl (derived from the datasheet). In the *Flow LSFM* mode, the sample is continuously moving along the tube at the programmed flow rate, but this movement is slow enough respect to the exposure time to avoid image blurring. In both *Classic* and *Flow LSFM* the light-sheet is created perpendicular to the optical table. In the *Hybrid LSFM* mode the scanning of the sample is performed by translation of the whole chamber containing the sample with a motor (PI M-501.1DG) through a fixed horizontal light sheet plane. Alternatively, the sample is kept static while the light sheet is scanned vertically and it is synchronized with the vertical movement of the detection objective, mounted on a piezo motor (PiezoConcept PiFoc). The imaging chamber includes a blue LED to permit brightfield imaging, and it enables the automatic detection of the passage of the sample under the FoV of the collection objective. Two Arduino controlled stepper motors (Nanotec, L4018S1204-M6) connected to the “imaging-tube” permit the rotation of the sample. The entire HT chamber is placed at 45 degrees respect to the illumination objectives. More information on the implementation of the high-throughput chamber can be found in Supplementary Fig. 4.

### Light-sheet thickness and illumination FoV measurements

The light-sheet thickness was determined by measuring the waist diameter from an image of a focused beam, at different aperture diameters (R1 to R5) which modulate the illuminating NA. The values are reported in Supplementary Fig. 2, and they were obtained as follow. The Gaussian beam appears propagating along the horizontal direction of the image and the intensity profile at its waist (perpendicularly to the propagation direction) was extracted through FIJI, and Gaussian fitted. The FWHM diameter, representing the LS thickness, was calculated from the standard deviation of the Gaussian fit as ${\mathrm{d}}_{\mathrm{waist}}={\mathrm{FWHM}}_{\mathrm{waist}}=2.355\upsigma$. From this value, the diameter of the beam at the Rayleigh range was calculated as ${\mathrm{d}}_{\mathrm{rayleigh}}=\sqrt{2}\cdot {\mathrm{d}}_{\mathrm{waist}}$. Through a custom-made FIJI macro, the intensity profile of each column of the image was extracted and sent to the FIJI built-in Gaussian fit, retrieving for each column the FWHM of the Gaussian peak as described above. This array of values represents the FWHM beam diameter as function of the propagating distance. The number of columns presenting values smaller than ${\mathrm{d}}_{\mathrm{rayleigh}}$ were counted and converted from pixel to micrometers (pixel size is 0.65 µm), delivering the illumination FoV.

### Resolution measurements

Lateral and axial resolutions obtained from two different detection objectives (Nikon 10x, 0.3 NA and Olympus 20x, 0.5 NA, leading to a pixel size of 433 nm and 195 nm respectively) were measured in two different mounting condition. In the first condition, sub-diffraction fluorescent beads (180 nm in diameter) were embedded in a 1.5% lmpa (low melting point agarose) cylinder, which is the usual mounting for the *Classic LSFM* configuration, and scanned across the light-sheet with a 100 nm z-step. In the second condition, in order to mimic the mounting used for the *Flow LSFM* and *Hybrid LSFM*, the same beads were inserted into a FEP tube (1.6 mm OD × 1 mm ID, the same used for the experiments on embryos). However, while in the real application no agarose is present in the FEP tube to constraint the sample, to maintain the beads steady during z-stack acquisition we embedded them into a low agarose concentration (0.5% lmpa) inside the FEP tube. See Supplementary Fig. 12 for the obtained values and mounting schematics. For each condition, 10 different beads were considered. The lateral resolutions were measured by retrieving the intensity profiles in both directions (x and y) and by fitting them through a Gaussian function. The FWHMs, corresponding to the actual resolutions, were calculated as ${R}_{lateral}={\mathrm{FWHM}}_{lateral}=2.355\upsigma$, were σ is the variance of the Gaussian fit. After reslicing in z the images of the same beads, the same procedure was applied to the z-axis profile, obtaining the axial resolutions as FWHM of the Gaussian fit.

### Environmental control

Environmental control is integrated into the 316L stainless steel imaging chamber for *Classic LSFM* modality. For temperature control, a heater (power resistor of 12 Ω and an aluminium plate) and a PT100 temperature sensor are adjacent to the bottom part of the chamber. These elements are connected to a PID controller (E5CN-H, Omron) which regulates the power delivery in order to maintain the temperature constant over time. Additionally, a NTC thermistor sensor (TSP-TH, Thorlabs) is inserted into the imaging medium and connected to a USB data logger (TSP01, Thorlabs). In this way, medium’s temperature, room’s temperature, and room’s humidity can be registered over time. A CO_2_ delivery system is also included from the top of the chamber, which is closed except for the space needed for sample insertion and translation. Finally, a refilling circuit permits to introduce a programmable amount of imaging medium during long term experiments, to compensate for eventual medium evaporation.

### Computer hardware and software

The Flexi-SPIM microscope feature a home-made software based on LabVIEW (National Instruments). This software permits the user to have access to the settings of the various devices, on a single graphic interface. The controller, and in consequence the settings and the regulation of imaging chamber, MPR, and shutters, have been integrated in this software. We run the experiments on a Dell Precision T7810 workstation, with 64 GB of RAM and equipped with a frame grabber (Active Silicon) for the sCMOS camera and digital-analogue card (National Instruments, PCIe-6363) providing up to 4 analogue outputs (two for the galvo and one for the piezo motor).

### Arduino-based controller

An Arduino UNO board is connected via USB to our workstation, and integrated in the LabVIEW software through the LIFA (LabVIEW Interface for Arduino) interface, provided by National Instrument. In order to control the four stepper motors (2 for the imaging chamber, and 2 for the MPR), the controller includes four Big Easy Drivers and two power supplies at + 12 V DC, 3.3 A. The controller also includes one power source at + 5 V DC, which supply the needed power for all the servomotors (MPR and shutters) and the LED (imaging chamber). More information in Supplementary Fig. 8.

### Zebrafish

All experiments involving zebrafish were carried in compliance with protocols approved by ICFO and by Comissió d'Experimentació Animal, Direcció General de Polítiques Ambientals i Medi Natural of the Departament de Territori i Sostenibilitat, Generalitat de Catalunya (Spain). "Neural activity recording in zebrafish larvae" (Expedient: FUE-2019-01161091; ID: PD8GZTVV7; Project: 10716).

### 3D cell cultures

For Supplementary Figure 6, complex 3D culture models embedded on alginate capsules including different combinations of tumour cell spheroids of non-small cell lung carcinoma (LC), cancer associated fibroblasts (CAF) and a monocytic cell line (THP-1) were generated following protocols described in^31^.

### Mouse embryos

All experiments involving mouse embryos were carried in compliance with protocols approved by the Comissió d'Experimentació Animal, Direcció General de Polítiques Ambientals i Medi Natural of the Departament de Territori i Sostenibilitat, Generalitat de Catalunya (Spain). "In vivo visualization of pre implantation process during mouse development through light-sheet fluorescence microscopy" (Expedient: FUE-2019-01190993; ID: XM5VTQ9BN; Project: 10758).

Animal procedures were conducted in accordance with standard ethical guidelines (European Communities Directive 2010/63/EU) and approved by the local ethical committees (Comissió d'Experimentació Animal, Direcció General de Polítiques Ambientals i Medi Natural of the Departament de Territori i Sostenibilitat, Generalitat de Catalunya (Spain)). The authors confirm that the study was carried out in compliance with the ARRIVE guidelines.

## Supplementary Information

Supplementary Information 1.

Supplementary Video 1.

Supplementary Video 2.

Supplementary Video 3.

Supplementary Video 4.

Supplementary Video 5.

Supplementary Video 6.

Supplementary Video 7.

## Data Availability

The datasets generated during the current study are available from the corresponding author on reasonable request.
