# Supplementary material for: Modular multimodal platform for classical and high throughput light sheet microscopy
Source: Sci Rep. 2022 Feb 4;12:1969. doi: 10.1038/s41598-022-05940-2 (PMC8817037; doi:10.1038/s41598-022-05940-2)
Supplement: Supplementary file 1 — Supplementary Information 1. [file 41598_2022_5940_MOESM1_ESM.pdf]

MODULAR MULTIMODAL PLATFORM FOR CLASSICAL AND HIGH THROUGHPUT LIGHT SHEET MICROSCOPY

Supplementary Material:

**Supplementary Figure 1**	General description of the Flexi-SPIM setup
**Supplementary Figure 2**	Description of the illumination path and DSLM module
**Supplementary Figure 3**	Description of the different detection paths
**Supplementary Figure 4**	High-Throughput Imaging Chamber with Rotation
**Supplementary Figure 5**	Bright-field and stack focusing
**Supplementary Figure 6**	HT analysis of co-cultured 3D samples
**Supplementary Figure 7**	High-Throughput screen of fixed immunostained zebrafish larvae
**Supplementary Figure 8**	Arduino based controller
**Supplementary Figure 9**	Multi-well plate reader
**Supplementary Figure 10**	Demonstration of the Raman LSFM using knife edge technique for spectral measurements.
**Supplementary Figure 11**	Orthogonal views of different samples acquired with the Flexi-SPIM setup
**Supplementary Figure 12**	Lateral and axial resolutions measurements
**Supplementary Note 1**	Control software and image processing tools
**Supplementary Table 1**	List of components of the different illumination schemes
**Supplementary Table 2**	List of components of the different detection schemes

**Supplementary Video 1**	*Classic LSFM* : zebrafish epiboly development
**Supplementary Video 2**	*Classic LSFM:* zebrafish development and fusion of the views
**Supplementary Video 3**	*Classic LSFM:* multicolour zebrafish development
**Supplementary Video 4**	*Flow LSFM:* entire zebrafish embryo scanning
**Supplementary Video 5**	*Hybrid LSFM:* macrophage movement at zebrafish head
**Supplementary Video 6**	*Hybrid LSFM:* macrophage recruitment at zebrafish caudal fin wound
**Supplementary Video 7**	*Hybrid LSFM:* zebrafish brain activity at 0.5 vol/sec

Supplementary Figure 1: General description of the Flexi-SPIM setup

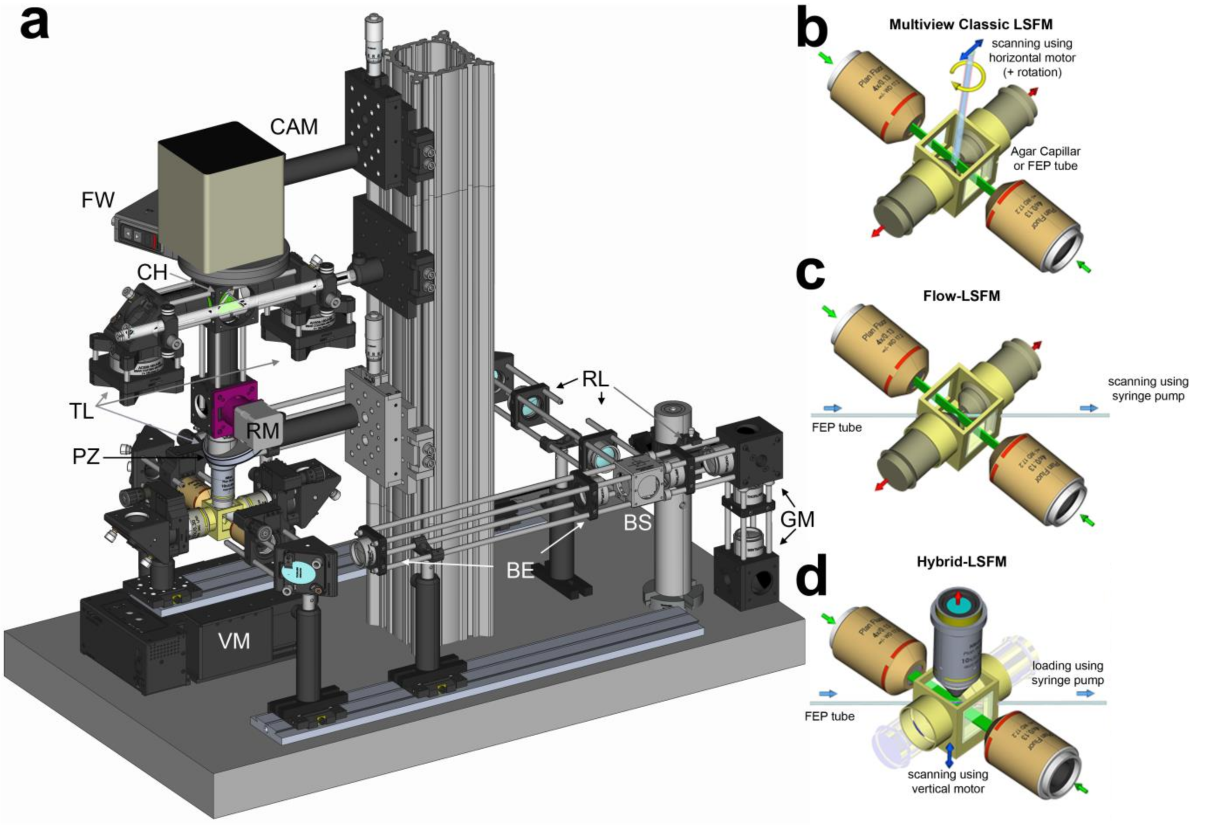

Supplementary Figure 1: General description of the Flexi-SPIM setup: (a) Schematic of the full system and submodules. The different elements are: **GM:** Galvo scan module; **BS:** Beam splitter for double side illumination; **RL:** Relay lenses; **BE:** Beam expander lenses; **VM:** Vertical motor; **TL:** Tube lenses; **CH:** Cube holder to select imaging mode; **FW:** Filter wheel; **CAM:** sCMOS camera; **PZ:** Piezo focus; **RM:** Raman-LSFM module. **(b-d)** Simplified schematic of the illumination/detection/sample scanning strategies of the different modes: **(b)** Multiview *Classic LSFM* mode allows *in toto* visualization of zebrafish embryos development with multicolour capabilities. **(c)** *Flow LSFM* mode allows simultaneous double side visualization of the sample as it flows through the light-sheet plane. **(d)** *Hybrid LSFM* mode allows multicolour high-throughput analysis of zebrafish and 3D cell cultures. Figure created with FreeCAD 0.16 (www.freecadweb.org).

Supplementary Figure 2: Description of the illumination path and DSLM module

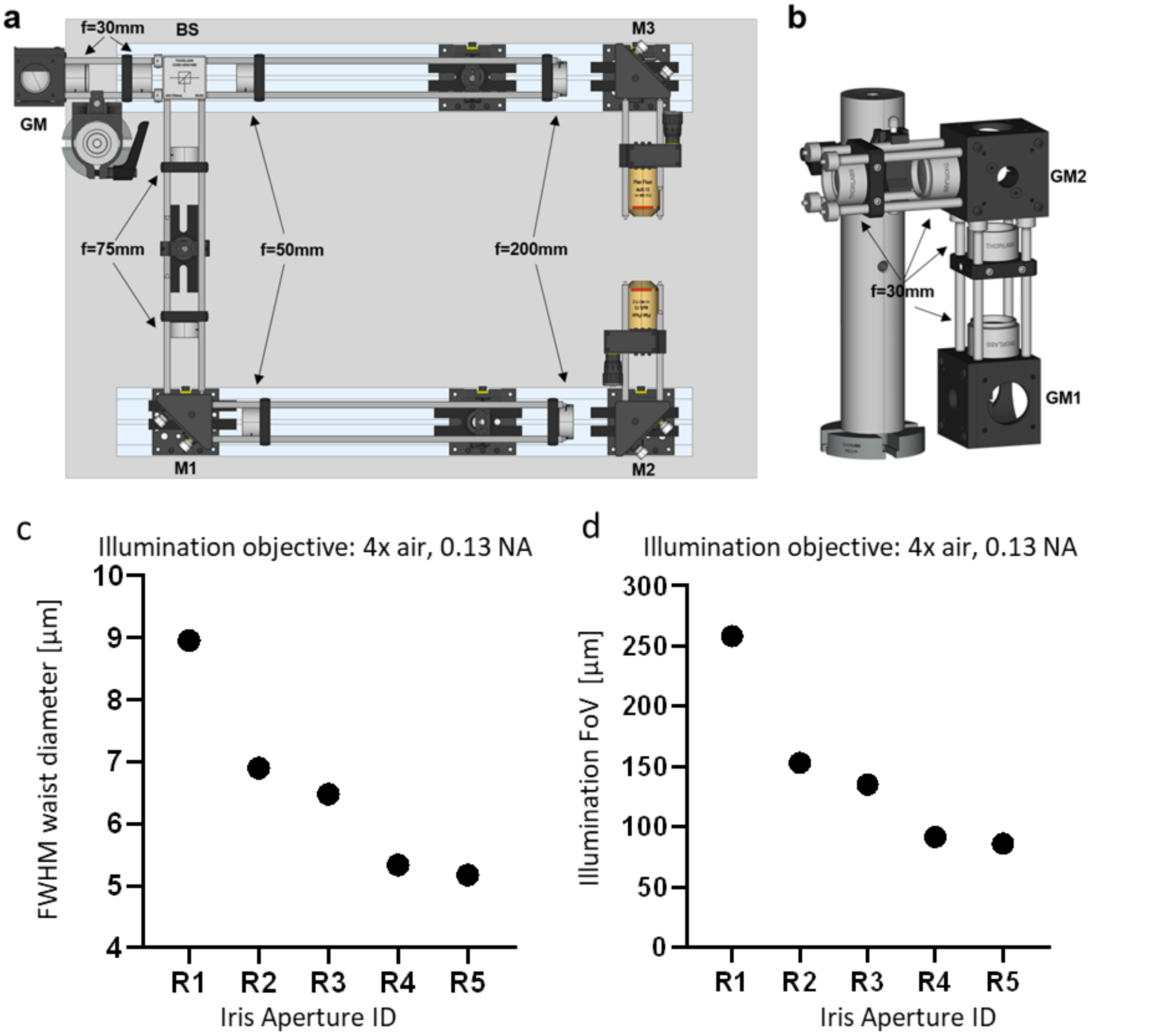

Supplementary Figure 2: Description of the illumination path and DSLM module. (a) Detailed scheme of the illumination arms with double side illumination. M: Mirror, BS: Beam splitter, GM: Galvo scanning module **(b)** DSLM attachment integrated by two conjugated galvo mirrors (GM), through four achromatic lenses (f=30 mm). GM1 is conjugated with GM2, and this one with the center of the beam splitter (BS). **(c)** The FWHM waist diameter of the illumination beam (i.e. the thickness of the light sheet) as a function of the iris aperture, defined by the index R1 (the smallest) to R5 (the largest). **(d)** The illumination FoV (i.e. the double of the Rayleigh range of a Gaussian beam) as a function of the iris aperture, defined by the index R1 (the smallest) to R5 (the largest). **(a-b)** created with FreeCAD 0.16 (www.freecadweb.org).

We have designed the Flexi-SPIM system so it can grow in complexity depending on the needs and the available budget. Its modular architecture allows scaling up the system from single side illumination to double side illumination, and from cylindrical lens (SPIM configuration) to galvanometric scanning (DSLM configuration) light-sheet generation. The components of the different modules are listed on **Supplementary Table 1**. Thorlabs cage system components are used to hold the optical elements. Although this choice increases the overall cost of the setup, it provides robustness to the system and simplifies the alignment procedure.

Compared with SPIM arrangement, DSLM configuration present several advantages. Among them, we highlight the simplified alignment procedure, the better use of the laser power, the possibility of advanced illumination schemes (Bessel beams, two-photon excitation, structured illumination, etc.) and possibility to implement the confocal line-scanning mode. Additionally, it allows the automatic

switch between two directions of the light sheet, parallel or perpendicular to the optical table, which is essential for the multimodality of the system. This can be also achieved in SPIM configuration, although in a manual manner, since cylindrical lenses are mounted on a manual rotational mount (CRM1). Moreover, both illumination and detection can be performed either simultanously either sequentially, multiplying the number of possible configurations.

It is important to notice that, due to the geometry of the system, depending whether if we generate the DSLM light-sheet parallel or perpendicular to the table, the dual side illumination will be synchronous or in opposite directions. This has a capital importance if we aim to implement the line confocal detection that will allow a reduced level of background signal in our images. In confocal line scanning, the laser beam is conjugated to the rolling shutter of the sCMOS camera, which acts as scanning slit. Hamamatsu cameras offer two rolling shutter modes. For fast scanning, reaching 100 fps full frame, the chip is divided in two and read out from center pixels towards periphery. Another mode, the light sheet mode, read outs the full chip in a single direction, thus reducing the overall speed to 33 fps. With our system, the first method could be implanted on the *Classic/Flow LSFM* mode, while the second could be applied to *Hybrid LSFM* mode. This will allow take full advantage of our systems capabilities.

The values reported in Supplementary Fig. 2c-d were obtained as follow. For each aperture diameter (R1 to R5), an image of the focused beam was acquired. The Gaussian beam appears propagating along the horizontal direction of the image. The FWHM beam diameter was calculated by Gaussian fitting the intensity profile of the beam (perpendicularly to the propagation direction) at its waist. The FWHM diameter, representing the LS thickness, was calculated from the standard deviation of the Gaussian fit as $\mathrm{d_{waist}} = \mathrm{FWHM_{waist}} = 2.355\ \sigma$. From this value, the diameter of the beam at the Rayleigh range was calculated as $\mathrm{d_{rayleigh}} = \sqrt{2} \cdot \mathrm{d_{waist}}$.

Through a custom-made FIJI macro, the intensity profile of each column of the image was extracted and sent to the FIJI built-in Gaussian fit, retrieving for each column the FWHM of the Gaussian peak as described above. This array of values represents the FWHM beam diameter as function of the propagating distance. The number of columns presenting values smaller than $\mathrm{d_{rayleigh}}$ were counted and converted from pixel to micrometers, delivering the illumination FoV.

Supplementary Figure 3: Description of the different detection path.

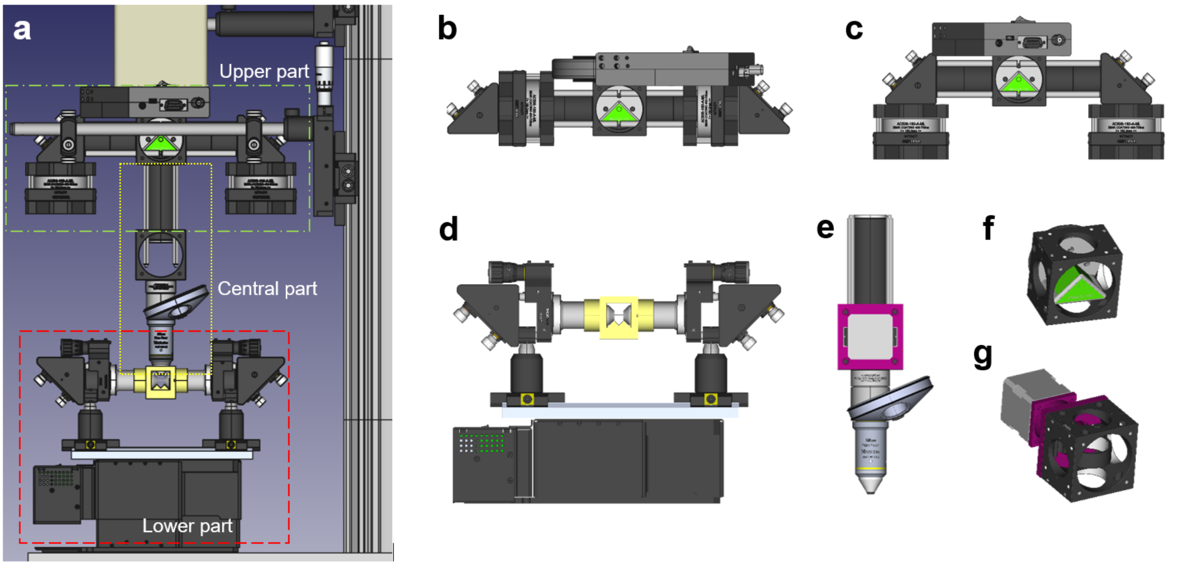

Detection → Illumination ↓	Olympus 10x W (NA 0.3)	Olympus 20x W (NA 0.5)	Nikon 10x W (NA 0.3)	Leica 20x W (NA 0.5)	Nikon 40x W (NA. 0.8)
Nikon 4x (NA 0.13)	Classic/Flow	Classic	Hybrid	Hybrid	X
Nikon 10x (NA 0.3)	X	Classic	X	Hybrid	Hybrid

Supplementary Figure 3: Scheme of the different elements in the detection path. (a) General overview of all the components (lower, central and upper parts) of the detection module. Upper part for **(b)** Nikon detection objectives (200 mm tube lenses) and **(c)** Olympus objectives (180 mm tube lenses). This part sustains the tube lenses, filter wheel and the central cube holder. **(d)** Lower part, consisting on a vertical motor, detection objectives and physiological chamber. **(e)** Attachment for Hybrid LSFM mode, holding the detection objective and the tube lens**.** Central cube holder containing a **(f)** knife-edge prism mirror and **(g)** motorized mirror, for simultaneous and sequencial dual side detection, respectively. The table shows the tested illumination/detection objective configurations. Figure created with FreeCAD 0.16 (www.freecadweb.org).

The main originality of the proposed setup can be found on the detection system. In order to provide multiple functionalities on the same microscope we have designed a modular platform that allows several acquisition modes, depending on the sample under analysis and the designed experiment. It basically contains three modules, a lower part for sample chamber holding; an upper part for dual view detection and a central module for upright detection. The parts of each module are listed in **Supplementary Table 2**.

The lower detection module consists in a rail mounted on top of a motorized vertical translation stage. This is needed to implement the *Hybrid LSFM* mode. In our case we used a PI-M501.1DG (Physics Instruments) although MLJ150 (Thorlabs) can be also used. For *Classic/Flow LSFM* mode, the rail can be screwed directly to the optical bench, or hold by the detection objective's mounts and rods. Attached to this rail several kinds of sample chambers can be easily inserted depending on acquisition mode chosen. We designed and tested chambers for different type of objective lenses, including water

dipping (Olympus, UMPLFLN10XW, NA 0.3; or UMPLFLN20XW, NA 0.5, Nikon 10x 0.3) and air objectives (Nikon 10x 0.3). Illumination is normally performed using air objectives, Nikon Plan Fluor 4x or Nikon 10x depending on the detection objective. Those objective lens are attached through cage assembly rods to the right-angle cage mount (Thorlabs, KCB1E) by manual translation mounts (Thorlabs, SM1Z), allowing fine focusing of the image.

For the upper part we have designed two variants of the detection scheme (**Supplementary Fig. 3(b-c)**). The chosen one will depend on the objective's brand used, since Nikon and Olympus present different parfocal distances and different tube lenses, of 200mm and 180 mm focal length respectively. When low magnification objectives are used, the sCMOS chip can be optically split in two parts using a knife-edge prism (Thorlabs, MRAK25-P01) inserted in the central cage cube (**Supplementary Fig. 3(f)**), so that each half visualizes simultaneously one side of the sample. For higher magnifications, alternate visualization of each side is performed with an Arduino controlled motorized mirror (Thorlabs PFR10-P01 on a Nanotec, L4018S1204-M6 stepper motor) (**Supplementary Fig. 3(g)**).

Alternatively, detection can be performed in an up-right configuration (**Supplementary Fig.3 (e)**) using water dipping objectives. An achromatic doublet with focal distance of 200 mm (AC254-200-A-ML) is used to form the image onto the camera chip. This module can be easily attached to the central cage cube of the upper module as shown in **Supplementary Fig 3(a)**, while removing the prism or motorized mirror.

Depending on the chosen modality, sample scanning is performed through different motors. When samples are mounted in the classical configuration, i.e. in agarose filled capillaries, the capillar is attached to the sample scanning system, composed of a linear stepper motor stage (Thorlabs, LNR25ZFS) for sample translation across the light-sheet plane and an Arduino controlled stepper motor (Nanotec, L4018S1204-M6) for sample rotation. When the *Flow LSFM* mode is used in order to increase throughput of the system, samples are loaded into FEP tubes and transported towards the detection objective field of view using a syringe pump (Tecan, Cavro Centris). In both cases the light-sheet is created perpendicular to the optical table. For the *Hybrid LSFM* mode the scanning of the sample is performed by translation of the chamber with a motor (PI M-501.1DG) through a fixed horizontal light sheet plane, or through a synchronized movement of the light-sheet (with a galvo) and the detection objective lens mounted on a piezo motor (PiezoConcept PiFoc), keeping the sample static.

Supplementary Figure 4: High-Throughput Imaging Chamber with Rotation

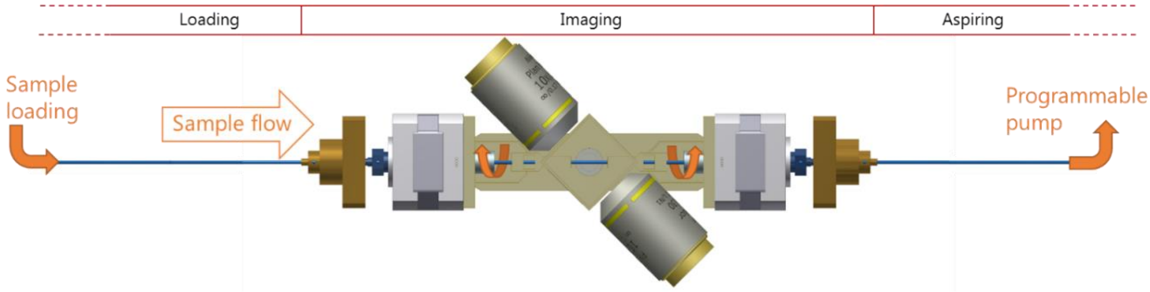

One of the main advantages given by the Flexi-SPIM setup is the straightforward sample mounting in the *Hybrid* configuration. As described, in this operational mode the sample is loaded into a FEP tube, which in turn is connected to a programmable syringe pump. This pump aspires the sample in a controlled manner until it reaches the field of view of the vertical detection objective. Here the imaging process takes place, with the possibility to scan the sample in the vertical direction. An imaging chamber is needed in order to: (i) maintain steady the FEP tube during the scanning; (ii) contain the water needed for the water-dipping detection objective; (iii) permit the rotation along the longitudinal axis of the sample; (iv) provide the system with quasi-brightfield imaging capability.

To accomplish these tasks, we designed and 3D printed a custom imaging chamber, and additional components that permits sample rotation and water-sealing. The 3D printed PLA model is the core part. It gives the structure to the entire HT chamber; it contains the imaging medium (water-based), and prevents its leaking. Centred on its longitudinal axis, a channel is present, through which the rotation FEP is placed. The PLA model features an almost-symmetric geometry: the central compartment contains the medium, and the detection objective is placed on its top. Its lateral walls are windows, glued with 22x22mm coverslips, to permit the illuminating light-sheet enter the chamber. Under this section, the model offers a void space for the insertion of a blue LED, which provides the illumination for the brightfield imaging. This LED is assured to the chamber's centre thanks to a 3D printed part, complementary to the void cavity. Light is delivered to the water-compartment thanks to a circular coverslip, glued with the 2-components UHU Endfest glue. Right outside the water-compartment and following the longitudinal channel, two small void cubicles contain some grease (High Vacuum Grease, DOW CORNING) which prevents the water leakage and helps the motion of the rotation FEP tube in the channel. At the opposite sides of the model, two vertical panels offer the possibility to attach and screw the two stepper motors (L4118S1404-M6X1 - NEMA 17, Nanotec Electronic GmbH & Co. KG). These motors offer a threaded (M6x1) hollow shaft and are driven by an Arduino-based controller, which gives the user the possibility to digitally control them. A custom-designed and internally machined part, called RotationScrew, is screwed to the rotator shaft of each stepper. The rotation FEP is fixed to it. Another mechanical part, called MotorConnector, is also internally produced and connected through 4x M3 screws to the motor's body. The MotorConnector offers a centred cavity where two o-rings (sized 3x3 and 4x3) and one bearing ball (623ZZ, ID=3mm, OD=10mm) are glued with the 2-components UHU Schnellfest glue. In the 3mm diameter remaining void cylinder, the tip of the RotationScrew is inserted. Both the MotorConnector and the RotationScrew feature a channel (ID 1.6mm) along the longitudinal direction. Finally, the FEP tube, carefully cut in three different parts (loading, rotation, and aspiring) is inserted in the central channel of the system. The first tip of the loading tube is reserved for the insertion of the sample. The other end is inserted in the left sided MotorConnector, for a length of 5mm, and fixed with the two retaining screws. This position corresponds to the interface between loading and rotation tube. Symmetrically, the first end of the aspiring tube is fixed with retaining screws in the right sided MotorConnector, and the other end is connected to the syringe pump. Between them, the rotation tube is present. It is fixed on both sides with retaining screws into the two RotationScrew components. From each RotationScrew, the rotation tube exceeds for a length of 10mm. The two tips of the rotation tube are in direct contact, inside the MotorConnector, with the loading and aspiring tubes. Figure created with Autodesk Inventor Professional 2015 (www.autodesk.com).

Supplementary Figure 5: Bright-field and stack focusing

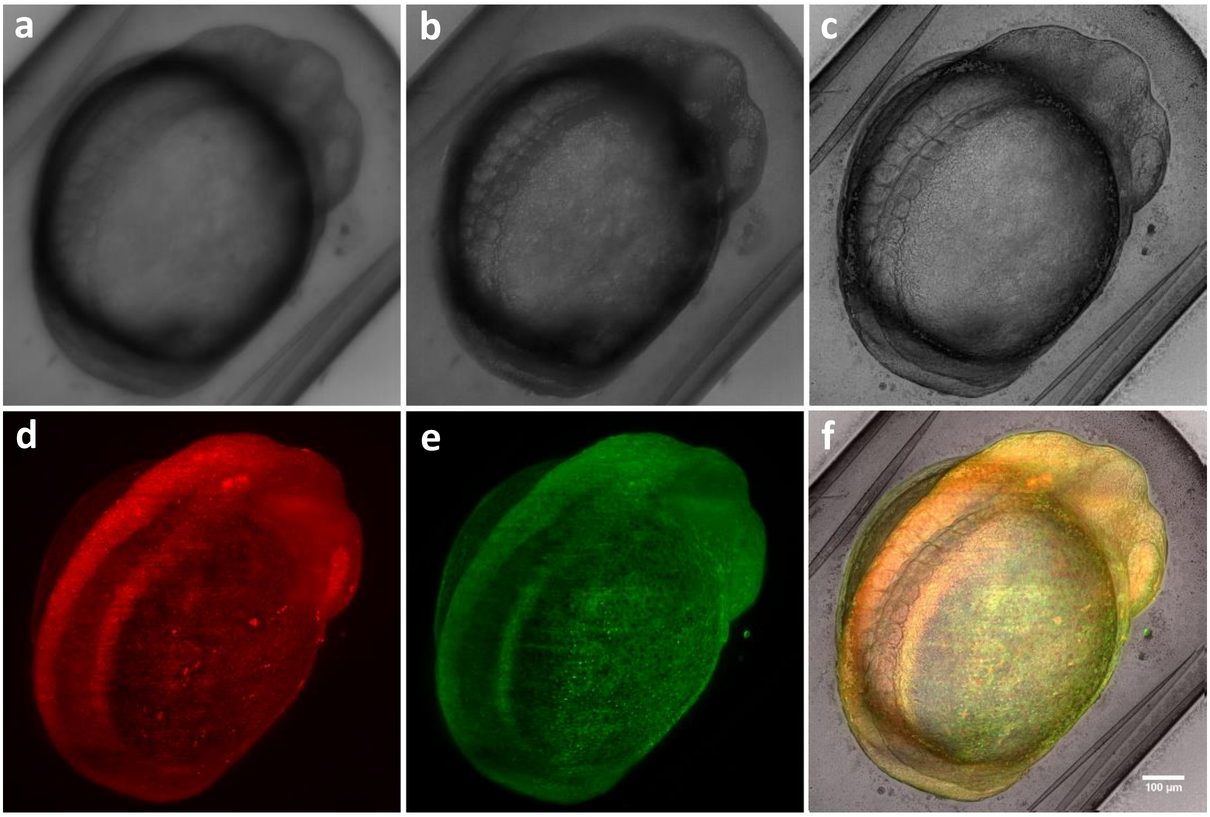

Supplementary Figure 5: Bright-field and stack focusing. The High-Throughput Imaging Chamber with Rotation developed for the *Hybrid LSFM* mode and described in Supplementary Fig. 4 includes a LED, which allow us to obtain bright-field images of the samples. However, for large samples only a portion of the specimen remains in focus of the detection objectives **(a)**. The system allows to scan the sample on the vertical axis (10 microns step) and create a better quality bright-field image by Maximum projection **(b)** or stack focusing **(c).** The images obtained can be finally be fused **(f)** with the corresponding channel of the light sheet fluorescent signal, obtaining complementary information and localizing the expression of the protein of interest. In the example we imaged a zebrafish embryo expressing a double transgenic line (GFP-dlck and RFP-myosin) **(d)** myosin (red) **(e)** tubulin (green). Scale bar 100 µm.

Supplementary Figure 6: HT analysis of co-cultured 3D samples

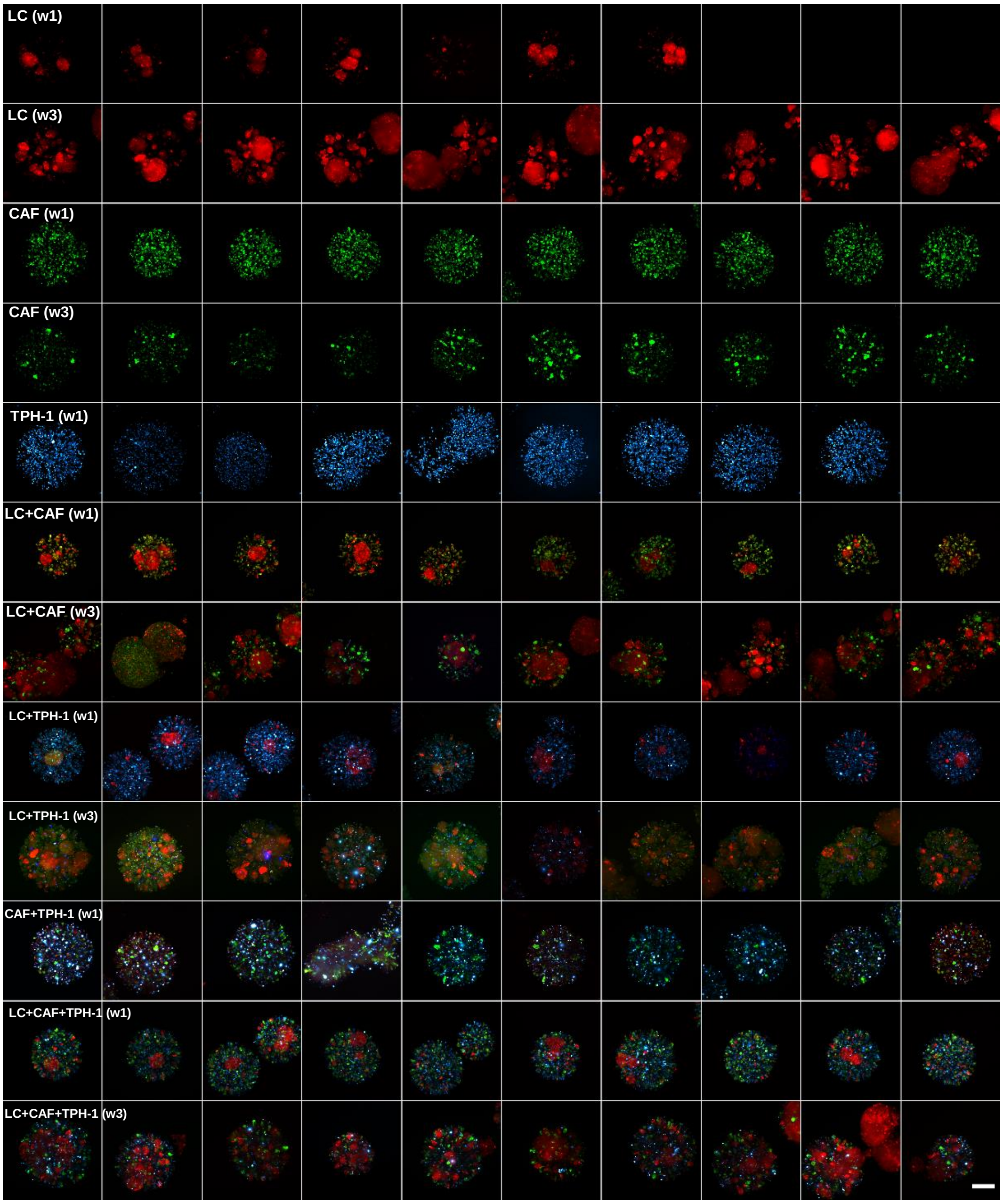

Supplementary Figure 6: HT analysis of co-cultured 3D samples. Different complex 3D culture models were evaluated in a semi-automatic manner using the *Hybrid LSFM* mode. During the experiment we image different 3D mono (rows 1-5), different 2 co-culture (rows 6-10) and 3 co-culture models (rows 11-12) including tumour cell spheroids of non-small cell lung carcinoma (LC) (tdTomato, red), cancer associated fibroblasts (CAF) (GFP, green) and a monocytic cell line (THP-1) (Cell tracker™, blue) on embedded on alginate capsules. For each group, except for monoculture of THP-1 (row 5) and co-culture of CAF-THP-1 (row 8), first row display results at week #1, while second row correspond to week #3. Scale bar 300 µm.

Supplementary Figure 7: High-Throughput screen of fixed immunostained zebrafish larvae

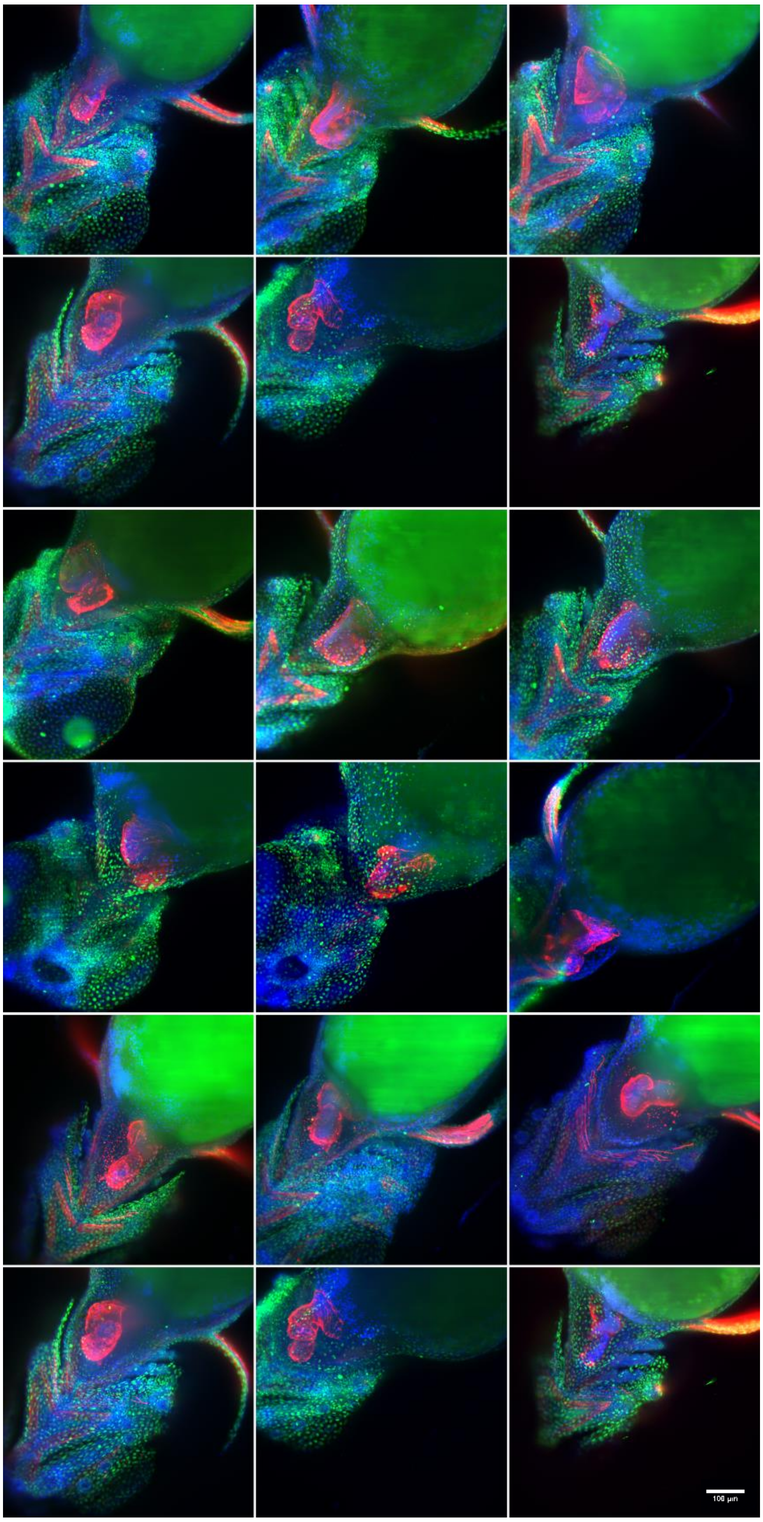

Supplementary Figure 7: High-Throughput screen of fixed immunostained zebrafish larvae. Multicolur maximum projection of eighteen 72hpf zebrafish larvae measured with the *Hybrid LSFM* mode with a 20x water dipping objective. Nuclei were stained with DAPI (blue), while myosin light chain (myl7) was conjugated with Alexa fluor 488 (red) and histone 3 acetylated in lysine 9 (H3K9Ac) was conjugated with Alexa Fluor 568 (green). Larvae pigmentation has been removed with 6% H_20_2. Zebrafish heart was reoriented towards the detection objective using the High-Throughput Imaging Chamber with Rotation. Scale bar 100 µm.

Supplementary Figure 8: Arduino based controller

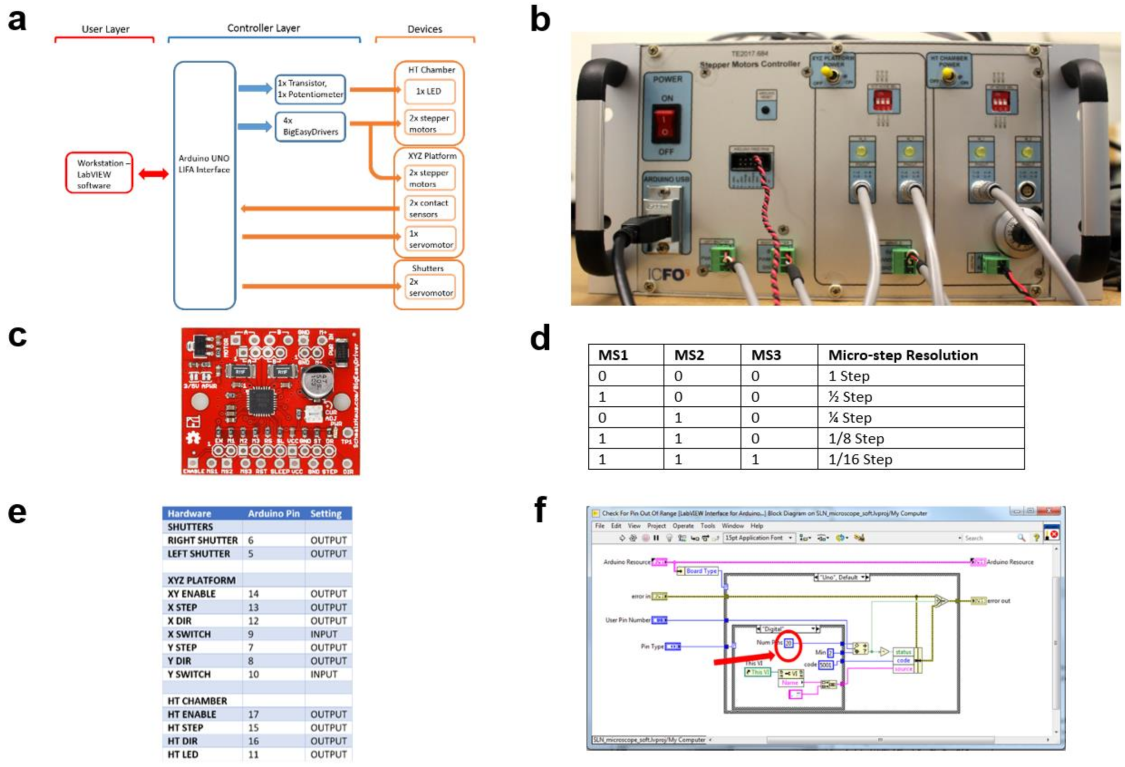

MS1	MS2	MS3	Micro-step Resolution
0	0	0	1 Step
1	0	0	½ Step
0	1	0	¼ Step
1	1	0	1/8 Step
1	1	1	1/16 Step

Hardware	Arduino Pin	Setting
SHUTTERS		
RIGHT SHUTTER	6	OUTPUT
LEFT SHUTTER	5	OUTPUT
XYZ PLATFORM		
XY ENABLE	14	OUTPUT
X STEP	13	OUTPUT
X DIR	12	OUTPUT
X SWITCH	9	INPUT
Y STEP	7	OUTPUT
Y DIR	8	OUTPUT
Y SWITCH	10	INPUT
HT CHAMBER		
HT ENABLE	17	OUTPUT
HT STEP	15	OUTPUT
HT DIR	16	OUTPUT
HT LED	11	OUTPUT

Supplementary Figure 8: Arduino based controller. (a) Principle schematic of the Arduino-controller application **(b)** Front panel of the Arduino-controller **(c)** Upper view of the Big Easy Driver **(d)** Micro-stepping resolution for given values of MS1, MS2, MS3 **(e)** Arduino-controller pinout **(f)** How to modify the default setting of the "*Check For Pin Out Of Range"* VI of the LIFA interface. **(a)** created with PowerPoint Office 2019 (www.microsoft.com).

The Flexi-SPIM setup features different hardware components that, thanks to a single LabVIEW-based homemade software, work in synergy. In order to interact with these components, we use an Arduino UNO board, which is connected via USB to our workstation, and is integrated in the LabVIEW software through the LIFA (LabVIEW Interface for Arduino) interface, provided by National Instrument. A principle schematic of the connections is shown in **Supplementary Fig. 8(a)**. The front panel layout of the controller is divided in 4 different areas, respect to the specific function and devices to be controlled, as shown in **Supplementary Fig. 8(b)**. These devices are:

- 1x stepper motor for sample rotation for *Classic LSFM* mode or tunable filter in *Raman LSFM* mode.
- 1x stepper motor for the alternate detection in *Classic LSFM* mode
- 2x servomotors used as shutters for the alternate-illumination configuration.
- 2x stepper motors and 1x LED of HT Chamber for *Hybrid LSFM* mode.
- 2x stepper motors, 1x servomotor and 2x switches that act as contact sensors for the multi-well plate reader platform.

The Arduino UNO board is the central part of the controller. Through its I/O pins, it provides the digital control of the devices. For a general stepper motor however, an additional driver is required, as the Arduino Board cannot provide the needed power supply. We choose the Big Easy Driver v1.2 (**Supplementary Fig. 8(c)**) from by Brian Schmalz (Schmalz Haus LLC).

The two motors relative to the HT Chamber share the same Arduino pins for ENABLE, STEP, and DIR. This assures that the two motors receive the same signal at the same time, moving synchronously. However, the connections between the two coils of the motors and the A and B pairs of pins of the

drivers are inverted between each other. In this way, the two motors move in opposite directions, as needed for the HT Chamber. The stepper motor drivers relative to the XYZ platform only share the ENABLE pin, as they supply two independent motors. During our experiment we always used the 1/16 micro-step resolution, which permits a smoother movement of the motor.

While the servomotors signal pins are directly connected to the output pins of the Arduino board, the LED is connected to a potentiometer and a MOSFET. Through the potentiometer (10 KΩ) we can regulate the correct intensity of the LED, while the transistor closes the circuit, and then powers the LED, when the GATE pin is triggered, through a digital output pin of the Arduino. Two Arduino digital pins are also used as input pins, to detect the status change of the switches implemented in the XYZ Platform, which indicate the Home position of the Platform. A resuming table of the Arduino pins needed is shown in **Supplementary Fig. 8(e)**. Additionally, some pins of the Arduino are left free, which could be further used for future development and integration (e.g. temperature sensor).

Thanks to the LIFA (LabVIEW Interface For Arduino) code, developed by National Instruments, we can communicate with the Arduino board through LabVIEW and dedicated VIs (Virtual Instruments). In our application we need only digital signals, thus we need to use some analogue pins of the Arduino board as digital pins. Although this is possible when programming the Arduino through its own text-based code, in LabVIEW we need to modify a default setting of the "*Check For Pin Out Of Range"* VI of the LIFA interface. In the "*Uno, Default*" case, "*Digital*" sub-case, changing the *Num Pins* constant to a value of 20 will let the usual analogue pins work as digital, as shown in **Supplementary Fig. 8(f).**

Supplementary Figure 9: Multi-well plate reader

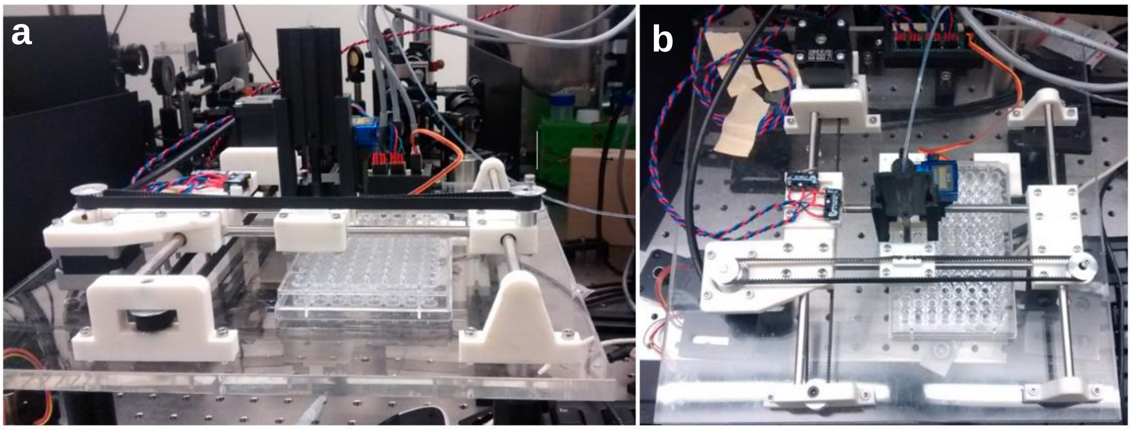

Supplementary Figure 9: Multiwell Plate reader (MPR) (a) Lateral view of the MPR **(b)** Top view of the MPR.

We implemented a multiwell plate reader (MPR) system, in order to automatize the loading procedure in the *Flow LSFM* and *Hybrid LSFM* modes. These imaging modalities, instead of using low melting point agarose, permits to bring the sample under the imaged field of view along a FEP tube, connected to a programmable syringe pump. The MPR system was adapted from an open-source project called XY Table (https://www.robotshop.com/letsmakerobots/xy-table) developed by Geir Andersen.

The main aim of the MPR is to automatize the loading process, thus increasing the number of samples imaged, for high-throughput application. It consists of 3D printed components connected through metallic rails. Two stepper motors provide the possibility to move the translational part in the horizontal (xy) plane, while a servomotor displaces in the vertical direction (z) the tip of the FEP tube. On the base of the platform, a 96-multiwell plate, commonly used in biological laboratories, is placed and fixed. The MPR is connected to the Arduino-based controller, which provides the power and signals to the motors, reads the two switches that sense the "home" position of the platform, and permits the customization and automation of the protocols. A custom-made LabVIEW interface allows easy control of all the elements of the MPR, as well the automation of complex protocols. In this way, the MPR is able to move the loading head above the multiwell plate in order to reach the well of interest, place the tip of the FEP tube into the selected well, and let the pump aspire the sample. Additionally, a webcam is placed under the transparent platform base, in order to detect if the loading of the sample has been successful. If not, force the MPR to repeat the sample loading protocol.

The MPR can also being used to implement more complex or diversified protocols, like loading and, after imaging, sorting of the sample in specific well, or even direct and automated drug delivery to specific wells.

Supplementary Figure 10: Demonstration of the Raman LSFM using kniffe edge technique for spectral measurements.

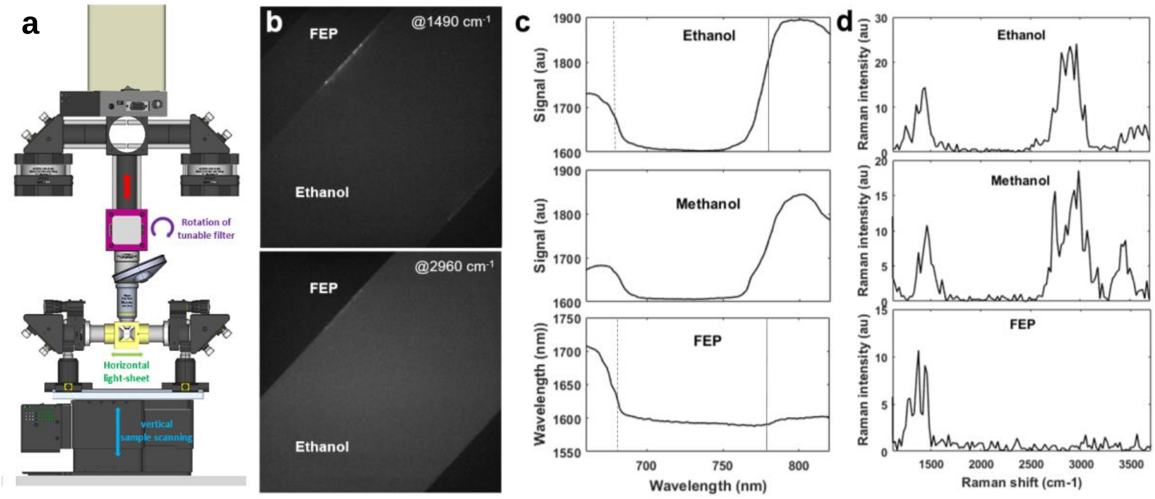

Supplementary Figure 10: Demonstration of the Raman LSFM using kniffe edge technique for spectral measurements. (a) Setup implementation in the Raman LSFM modality. The setup includes an interferometric tunable filter motorized using an Arduino controlled stepper motor (magenta). **(b)** Spectrally resolved Raman images at 1490cm^{-1} (upper panel) and at 2960 cm^{-1} (lower panel). **(c)** Spectral knife-edge traces and **(d)** derivatives of ethanol (upper panel), methanol (middle panel) and FEP (Fluorinated Ethilene Propilene) (lower panel). **(a)** created with FreeCAD 0.16 (www.freecadweb.org).

Supplementary Figure 11: Orthogonal views of different samples acquired with the Flexi-SPIM setup.

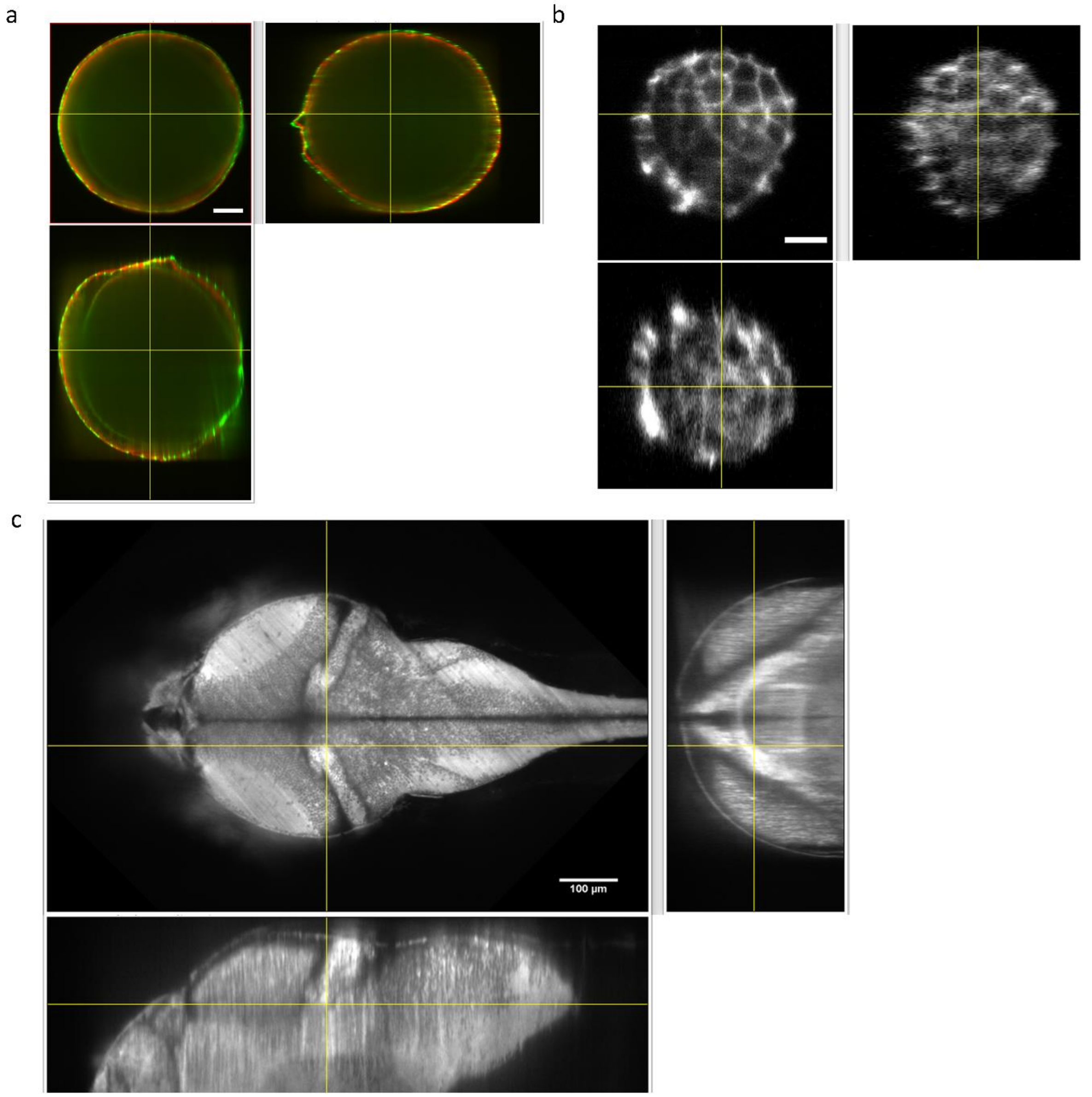

Supplementary Figure 11: Orthogonal views of different samples acquired with the Flexi-SPIM setup. (a) Zebrafish embryo displayed in Figure 3E showing actin (green) and tubulin (red). Acquired with *Classic LSFM* with double illumination/detection scheme. Scale bar 100 µm. **(b)** Mouse embryo (E-Cadherin-GFP) displayed in Figure 3G at about E4.5 stage visualized through *Classic LSFM* with double illumination/detection scheme. Scale bar 20 µm. **(c)** Zebrafish larvae expressing GCamP6 panneuronally displayed in Figure 5L, acquired with the *Hybrid LSFM* and piezo focus control. Scale bar 100 µm.

Supplementary Figure 12: Lateral and axial resolutions measurements.

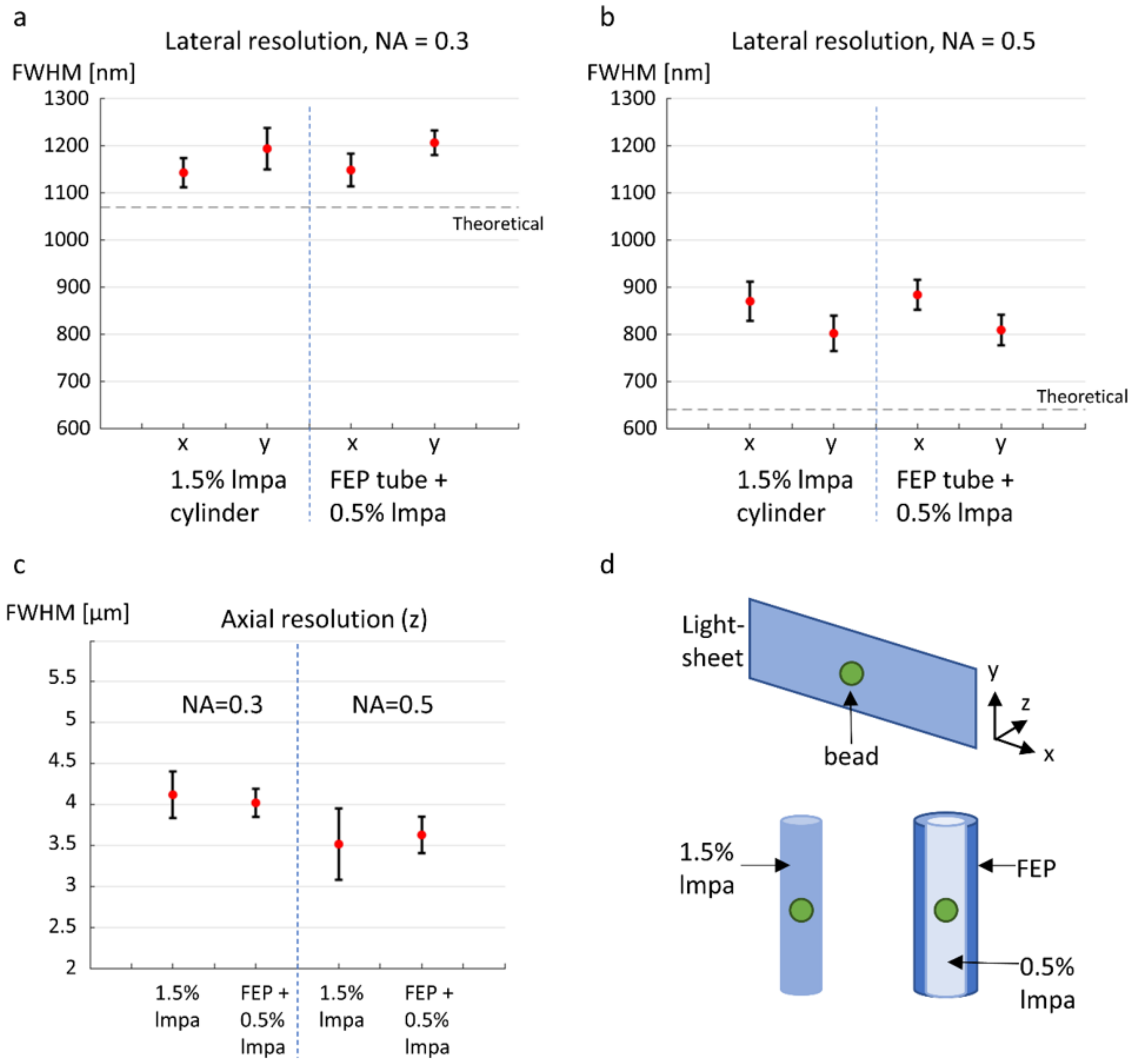

Supplementary Figure 12: Resolution measurements with different mounting configurations. (a) The lateral resolution measured along (x) and perpendicularly (y) to the light-sheet propagation direction through a 0.3 NA objective (pixel size = 433 nm). FWHM values are obtained by imaging of sub-diffraction beads embedded in a 1.5% lmpa (low melting point agarose) cylinder (left) and in 0.5% lmpa within a 1.6mm OD x 1mm ID FEP tube. The dotted line represents the theoretical resolution, which equals 1067.5 nm. **(b)** The lateral resolution measured through a 0.5 NA objective (pixel size = 195 nm) with the same conditions as (a). The dotted line represents the theoretical resolution, which equals 640.5 nm. **(c)** Axial FWHM values obtained for the same conditions as in **(a)**, for 0.3 and 0.5 NA objectives. The shown values are the average FWHMs, error bars represent ± standard deviations, N=10 beads for each condition. **(d)** Schematic of the coordinate system and of the mounting solutions of the beads for these measurements. Note that in the real application, when using the FEP tube mounting, no agarose embedding is needed. However, in order to maintain the beads steady during imaging, they were embedded in 0.5% lmpa. As shown, the introduction of the embedding medium between the objective and the sample (either lmpa or FEP tube) slightly degrade the resolutions with respect to the theoretical ones, but do not interfere with the capability of imaging at cellular levels. **(d)** created with PowerPoint Office 2019 (www.microsoft.com).

Supplementary Note 1: Control software and image processing tools

The Flexi-SPIM microscope offers a high automation degree, letting also non-microscope-specialists being able to use all its functions. This has been possible thanks to the home-made LabVIEW software, which controls almost all the aspects in a user friendly graphic interface.

The software is mainly divided in two layers: the first is the “LIVE” layer. Through the graphical front panel, the user can directly act on the different components. This layer include all the controls, and it is used to load the sample, position it, acquire snapshots, receive feedbacks on the status of the devices, define the light sheet parameters through the galvo mirrors, and regulate the best proprieties for the acquisition (e.g. exposure time, camera’s ROI, laser power, etc.). Different functions are separated in different tabs, providing an intuitive organization. On the block diagram, the software provides the initialization of all the connected devices (camera, motors, Arduino controller, galvo mirrors, syringe pump, filter wheel, lasers) and then will execute two parallel loops. The first one is entirely dedicated to the camera settings, and to the displaying of the current detected image. The second one manages the actions to be taken in the case the user changes any of the parameters on the other devices, or the protocols. This permits to visualize the “live” image and permits to define the best parameters for the automated acquisition.

The second layer of the software consist of a set of so-called subVIs. For each of the operational modes of the microscopes at the hardware level in fact, a number of different protocols can be implemented, e.g. simultaneous or alternate illumination, simultaneous or alternate detection, translation or rotation of the sample during imaging, etc. Thus, each subVI corresponds to a different acquisition mode. Once the settings are ready for the imaging the first layer of the software, based on the parameters inserted by the user, passes the control of the hardware to one of these subVI, which performs the actual imaging procedure and save the acquired data and related metadata file.

The different acquisition modes could be resumed as follow:

For *Classic LSFM*:

- Simultaneous double illumination and simultaneous double detection;
- Simultaneous double illumination and alternated double detection;
- Alternated double illumination and simultaneous double detection;
- Alternated double illumination and alternated double detection;

For *Flow LSFM*:

- Simultaneous double illumination and simultaneous double detection

For *Hybrid LSFM*:

- Simultaneous double illumination and single detection.
- Alternate double illumination and single detection.
- Fast scanning (up to 1 volume per second containing 50 planes) using the PiFoc system.
- Brightfield
- Raman spectra acquisition.

We used the open source Fiji[1] software to process all the acquired data. For image fusion and stitching we used “Pairwise stitching”[2] plugin, for image registration “StackReg”[3] plugin, “Gaussian_Stack_Focuser”[4] plugin for bright-field stack focusing, and “TransformJ”[5] for image shearing.

Supplementary Table 1: List of components of the different illumination schemes

ILLUMINATION SINGLE SIDE (x2 if double side)			
	**Reference**	**Description**	**Quantity**
**OPTICS**	AC254-050-A-ML	f=50 mm, Ø1" Achromatic Doublet, SM1-Threaded Mount, ARC: 400-700 nm	1
	AC254-200-A-ML	f=200 mm, Ø1" Achromatic Doublet, SM1-Threaded Mount, ARC: 400-700 nm	1
	PFE10-P01	1" Protected Silver Elliptical Mirror, 450 nm - 20 µm	1
**CAGE SYSTEM**	KCB1E/M	Right-Angle Kinematic Elliptical Mirror Mount with Tapped Cage Rod Holes, 30 mm Cage System and SM1 Compatible, M4 and M6 Mounting Holes	1
	SM1Z	Z-Axis Translation Mount, 30 mm Cage Compatible	1
	CP08/M	SM1 Threaded 30 mm Enhanced Clamping Cage Plate, 0.35" Thick, M4 Tap	2
	CP02B	30 mm Cage Mounting Bracket	1
	ER12	Cage Assembly Rod, 12" (304.8 mm) Long, Ø6 mm	4
	ER4	Cage Assembly Rod, 4" (101.6 mm) Long, Ø6 mm	4
**MOUNTS**	XT66SP-500	66 mm Single Dovetail Rail, L = 500 mm	1
	XT66P2/M	Rail Carriage for 66 mm Rails with M4 & M6 Taps	2
	TR50/M	Ø12.7 mm Optical Post, SS, M4 Setscrew, M6 Tap, L = 50 mm	2
	BA1	Mounting Base, 1" x 3" x 3/8"	2
	PH100/M	Ø12.7 mm Post Holder, Spring-Loaded Hex-Locking Thumbscrew, L=100 mm	2

Table 1.1: Components of the illumination arm for a single side illumination. If double side is implemented, double the number of elements and add the components in the Bridge Table.

BRIDGE			
	**Reference**	**Description**	**Quantity**
**OPTICS**	AC254-075-A-ML	f=75 mm, Ø1" Achromatic Doublet, SM1-Threaded Mount, ARC: 400-700 nm	2
	PFE10-P01	1" Protected Silver Elliptical Mirror, 450 nm - 20 µm	3
	CCM1-BS013/M	30 mm Cage Cube-Mounted Non-Polarizing Beamsplitter, 400 - 700 nm, M4 Tap	1
**CAGE SYSTEM**	KCB1E/M	Right-Angle Kinematic Elliptical Mirror Mount with Tapped Cage Rod Holes, 30 mm Cage System and SM1 Compatible, M4 and M6 Mounting Holes	1
	CP08/M	SM1 Threaded 30 mm Enhanced Clamping Cage Plate, 0.35" Thick, M4 Tap	2
	CP02B	30 mm Cage Mounting Bracket	1
	ER3	Cage Assembly Rod, 3" (76.2 mm) Long, Ø6 mm	2
	ER10	Cage Assembly Rod, 10" (254.0 mm) Long, Ø6 mm	3
**MOUNTS**	TR50/M	Ø12.7 mm Optical Post, SS, M4 Setscrew, M6 Tap, L = 50 mm	2
	BA1	Mounting Base, 1" x 3" x 3/8"	2
	PH100/M	Ø12.7 mm Post Holder, Spring-Loaded Hex-Locking Thumbscrew, L=100 mm	2

Table 1.2: Components of the bridge between opposite illumination arms.

SPIM ILLUMINATION-SINGLE SIDE (x2 if double side)			
	**Reference**	**Description**	**Quantity**
**OPTICS**	ACY254-150-A	f = 150 mm, Ø1" Cylindrical Achromat, AR Coating: 350 - 700 nm	1
**CAGE SYSTEM**	GCM001	Cage Rotation Mount for Ø1" Optics, SM1 Threaded, M4 Tap	1
	ER2	Cage Assembly Rod, 2" Long, Ø6 mm	4
	VA100C/M	30 mm Cage System Adjustable Slit, M4 Tap, Metric Micrometer	1

Table 1.3: Components of the module for SPIM illumination configuration.

DSLM-SCAN SYSTEM			
	**Reference**	**Description**	**Quantity**
**OPTICS**	AC254-030-A-ML	f=30 mm, Ø1" Achromatic Doublet, SM1-Threaded Mount, ARC: 400-700 nm	4
	GVSM002/M	2D Galvo System with Metric Accessories	1
**CAGE SYSTEM**	GCM001	1D Galvo 30 mm Cage System Mount	2
	CP08/M	SM1 Threaded 30 mm Enhanced Clamping Cage Plate, 0.35" Thick, M4 Tap	2
	ERSCA	Rod Adapter for Ø6 mm ER Rods, Qty. 1	8
	ER3	Cage Assembly Rod, 3" (76.2 mm) Long, Ø6 mm	8
	ER025	Cage Assembly Rod, 1/4" (6.4 mm) Long, Ø6 mm	8
	CH1530/M	30 mm Cage Clamp for Ø1.5" Posts, Included Quick-Release Handle, Metric	1
**MOUNTS**	P200/M	Ø1.5" Mounting Post, M6 Taps, L = 200 mm	1
	PB2/M	Metric Mounting Post Base, Ø61 mm x 12.7 mm Thick	1
	SM1T2	SM1 (1.035"-40) Coupler, External Threads, 0.5" Long	2

Table 1.4: Components of the scanner module for DSLM illumination configuration.

Supplementary Table 2: List of components of the different detection schemes

DETECTION LOWER PART			
	**Reference**	**Description**	**Quantity**
**CAGE SYSTEM**	ER1	Cage Assembly Rod, 1" (25.4 mm) Long, Ø6 mm	8
	CP02B	30 mm Cage Mounting Bracket	2
**MOUNTS**	KCB1E/M	Right-Angle Kinematic Elliptical Mirror Mount with Tapped Cage Rod Holes, 30 mm Cage System and SM1 Compatible, M4 and M6 Mounting Holes	2
	XT66P2/M	Rail Carriage for 66 mm Rails with M4 & M6 Taps	3
	XT66SP-500	66 mm Single Dovetail Rail, L = 500 mm	1
	PH30/M	12.7 mm Post Holder, Spring-Loaded Hex-Locking Thumbscrew, L=30 mm	2
	TR1	Ø1/2" Optical Post, SS, 8-32 Setscrew, 1/4"-20 Tap, L = 1"	2
	SM1Z	Z-Axis Translation Mount, 30 mm Cage Compatible	2
	SM1V10	Ø1" Adjustable Lens Tube, 0.81" Travel Range	2
	SM1A3TS	thermally Insulating Adapter with External SM1 Threads and Internal RMS Threads	2

Table 2.1: Components of the lower part of the detection arm. This module holds the detection objectives of the system.

DETECTION UPPER PART			
	**Reference**	**Description**	**Quantity**
**OPTICS**	AC508-180-A-ML	f=180 mm, Ø2" Achromatic Doublet, SM2-Threaded Mount, ARC: 400-700 nm	2
	AC508-200-A-ML	**Alternatively for Nikon Objectives** f=200 mm, Ø2" Achromatic Doublet, SM2-Threaded Mount, ARC: 400-700 nm	2
**CAGE SYSTEM**	CFW6	30 mm Cage Filter Wheel for Six Ø1" Filters	1
	C6W	30 mm Cage Cube, Ø6 mm Through Holes	1
	ER2	Cage Assembly Rod, 2" (50.8 mm) Long, Ø6 mm	12
	ER3	Cage Assembly Rod, 3" (76.2 mm) Long, Ø6 mm	4
	ER1	Cage Assembly Rod, 1" (25.4 mm) Long, Ø6 mm	2
	ER025	Cage Assembly Rod, 1/4" (6.4 mm) Long, Ø6 mm	4
	LCP02/M	30 mm to 60 mm Cage Plate Adapter, M4 Tap	4
	LCP01/M	60 mm Cage Plate, SM2 Threaded, 0.5" Thick, M4 Tap (Two SM2RR Retaining Rings Included)	2
**MOUNTS**	SM1L10	SM1 Lens Tube, 1.00" Thread Depth, One Retaining Ring Included	4
	KCB1E/M	Right-Angle Kinematic Elliptical Mirror Mount with Tapped Cage Rod Holes, 30 mm Cage System and SM1 Compatible, M4 and M6 Mounting Holes	2

Table 2.2: Components of the upper part of the detection arm. This module holds tube lenses for *Classic/Flow LSFM* modes. It also includes the central cage cube, that is the central part of our design and allow attaching the different submodules described in Supplementary Fig. 2.

DETECTION CENTRAL PART			
	**Reference**	**Description**	**Quantity**
**OPTICS**	AC254-200-A-ML	f=200 mm, Ø1" Achromatic Doublet, SM1-Threaded Mount, ARC: 400-700 nm	2
**CAGE SYSTEM**	C6W	30 mm Cage Cube, Ø6 mm Through Holes	2
	ER4	Cage Assembly Rod, 4" (101.6 mm) Long, Ø6 mm	4
	CP360R/M	Pivoting, Quick-Release, Ø1" Optic Mount for 30 mm Cage System (Metric)	1
**MOUNTS**	OT1	Objective Lens Turret, 4 RMS-Threaded Objective Ports	1
	SM1S25	SM1 Lens Tube Spacer, 2.5" Long	1
**MOTOR**		Motorized Lab Jack (Physics Instrumente)	1
	MLJ050/M	**Alternatively** Motorized Lab Jack, 50.8 mm Travel, M6 Taps	1

Table 2.3: Components of the central part of the detection arm. This module holds the objective and tube lenses for *Hybrid LSFM* modes. A second cage cube helps to integrate the *Raman LSFM* mode into the system.

COLUMN PART			
	**Reference**	**Description**	**Quantity**
**MOUNTS**	XT95-500	95 mm Construction Rail, L = 500 mm	1
	XT95P11/M	Drop-On Rail Carriage for 95 mm Rails, M6 Tapped Holes	2
	PT1/M	25 mm Translation Stage with Standard Micrometer, M6 Taps	2
	PH30/M	12.7 mm Post Holder, Spring-Loaded Hex-Locking Thumbscrew, L=30 mm	1
	PH100/M	Ø12.7 mm Post Holder, Spring-Loaded Hex-Locking Thumbscrew, L=100 mm	1
	TR300/M	Ø12.7 mm Optical Post, SS, M4 Setscrew, M6 Tap, L = 300 mm	1
	TR40/M	Ø12.7 mm Optical Post, SS, M4 Setscrew, M6 Tap, L = 40 mm	3
	RA90/M	Right-Angle Clamp for Ø1/2" Posts, 5 mm Hex	2

Table 2.4: Components of the supporting structure of the detection upper part of the detection arm. This module holds the camera and upper detection part previously described.

OBJECTIVES			
	**Thorlabs Reference**	**Description**	**Quantity**
**OPTICS**	N4X-PF	4X Nikon Plan Fluorite Imaging Objective, 0.13 NA, 17.2 mm WD	2
	N10X-PF	10X Nikon Plan Fluorite Imaging Objective, 0.3 NA, 16 mm WD	2
		10X Olympus UMPlanFLN, 0.30 NA, 3.5 mm WD	2
		20X Olympus UMPlanFLN, 0.50 NA, 3.5 mm WD	2
	N10XW-PF	10X Nikon CFI Plan Fluorite Objective, 0.30 NA, 3.5 mm WD	1
	N40X-NIR	40X Nikon CFI APO NIR Objective, 0.80 NA, 3.5 mm WD	1
		20X Leica, 0.50 NA, 3.5 mm WD	1

Table 2.5: List of objectives available and tested on the Flexi-SPIM system.

Supplementary Video 1: Zebrafish embryo development during epiboly visualized through the *Classic LSFM* mode. Microtubules are labels through dclk:GFP transgenic line. The four views correspond to the maximum intensity projections for the double illumination/double detection scheme.

Supplementary Video 2: Development of a zebrafish embryo expressing GFP:actin visualized through the *Classic LSFM*. **(a)** Both sides of the sample are acquired simultaneously with a single camera, after the two detection views are fused into a single image in the knife-edge mirror. In this example, illumination is performed simultaneously from both sides. **(b)** The two detected views can be easily fused into a single dataset that preserve the more relevant information of each view. **(c)** Colour depth map representation of the previous fused dataset.

Supplementary Video 3: Maximum intensity projection of a GFP:Myosin zebrafish embryo development (gray), with injected mCherry:Myosin bleb cells (orange). Images were acquired with the *Classic LSFM* mode, with simultaneous dual side illumination and dual side detection, allowing to visualize the whole embryo sphere. The total movie consists of 150 time points every 2 minutes.

Supplementary Video 4: Using the *Flow LSFM* mode, an entire Fli-GFP zebrafish embryo can be visualized as it flows through the system. The front view (top panel) and the back view (middle panel) are simultaneously registered on the same camera chip. The two views can be subsequently fused together (bottom panel).

Supplementary Video 5: Maximum intensity projection of macrophage migration on the zebrafish head. The sample was monitored using the *Hybrid LSFM* mode, including sequential dual side illumination and two channel acquisitions, autofluorescence (green filter) and macrophage expressing arg2:mCherry (red filter) (upper panel). For each time point, four different 100 images stacks were acquired at 10 fps (100 ms exposure time) over a range of 300 microns (z_{step}=3 microns). In the lower panel only macrophages are displayed. The total movie consists of 50 time points every 10 minutes.

Supplementary Video 6: Maximum intensity projection of the zebrafish caudal fin expressing arg2:mCherry after provoking a wound at the tail. Macrophages were monitored with the *Hybrid LSFM* mode with sequential dual side illumination and two channel acquisitions at high speed (0.5 vol/sec, with piezo refocusing). The total movie consist in 134 time points every 2 minutes. For each time point, four different 100 images stacks were acquired at 50 fps (20 ms exposure time) over a range of 200 microns (z_{step}=2 microns). Here only red channel is displayed.

Supplementary Video 7: Zebrafish brain activity monitored in 6 days post-fertilization larvae expressing GCamP6s pan-neuronally with the *Hybrid LSFM mode* and piezo refocusing at 1 vol/sec, using a triangular wave. In this movie we only display one direction of the scanned wave, i.e. recorded at 0.5 vol/sec: slow motion visualization of the brain volume; a single plane time-lapse (one image every 2 second); the maximum intensity projection time-lapse; depth colour coded maximum intensity projection. For each time point, 50 images stacks were acquired at 100 fps (10 ms exposure time) over a range of 300 microns (z_{step}=6 microns).

References

1. Schindelin, J. *et al.* Fiji: an open-source platform for biological-image analysis. *Nat. Methods* **9**, 676–682 (2012).

2. Preibisch, S., Saalfeld, S. & Tomancak, P. Globally optimal stitching of tiled 3D microscopic image acquisitions. *Bioinformatics* **25**, 1463–1465 (2009).

3. Thévenaz, P., Ruttimann, U. E. & Unser, M. A pyramid approach to subpixel registration based on intensity. *IEEE Trans. image Process. a Publ. IEEE Signal Process. Soc.* **7**, 27–41 (1998).

4. Stack Focuser. https://imagej.nih.gov/ij/plugins/stack-focuser.html.

5. Meijering, E. H., Niessen, W. J. & Viergever, M. A. Quantitative evaluation of convolution-based methods for medical image interpolation. *Med. Image Anal.* **5**, 111–126 (2001).
